# Enabling low cost biopharmaceuticals: high level interferon alpha-2b production in *Trichoderma reesei*

**DOI:** 10.1186/s12934-016-0508-5

**Published:** 2016-06-10

**Authors:** Christopher P. Landowski, Eero Mustalahti, Ramon Wahl, Laurence Croute, Dhinakaran Sivasiddarthan, Ann Westerholm-Parvinen, Benjamin Sommer, Christian Ostermeier, Bernhard Helk, Juhani Saarinen, Markku Saloheimo

**Affiliations:** VTT Technical Research Centre of Finland Ltd., Espoo, Finland; Novartis Pharma AG, Basel, Switzerland; Glykos Finland Oy, Helsinki, Finland

**Keywords:** *Trichoderma reesei*, Filamentous fungi, Interferon, Therapeutic proteins, Proteases, Cytokines

## Abstract

**Background:**

The filamentous fungus *Trichoderma reesei* has tremendous capability to secrete over 100 g/L of proteins and therefore it would make an excellent host system for production of high levels of therapeutic proteins at low cost. We have developed *T. reesei* strains suitable for production of therapeutic proteins by reducing the secreted protease activity. Protease activity has been the major hindrance to achieving high production levels. We have constructed a series of interferon alpha-2b (IFNα-2b) production strains with 9 protease deletions to gain knowledge for further strain development.

**Results:**

We have identified two protease deletions that dramatically improved the production levels. Deletion of the subtilisin protease *slp7* and the metalloprotease *amp2* has enabled production levels of IFNα-2b up to 2.1 and 2.4 g/L, respectively. With addition of soybean trypsin protease inhibitor the level of production improved to 4.5 g/L, with an additional 1.8 g/L still bound to the secretion carrier protein.

**Conclusions:**

High levels of IFNα-2b were produced using *T. reesei* strains with reduced protease secretion. Further strain development can be done to improve the production system by reducing protease activity and improving carrier protein cleavage.

**Electronic supplementary material:**

The online version of this article (doi:10.1186/s12934-016-0508-5) contains supplementary material, which is available to authorized users.

## Background

Interferon alpha-2b (IFNα-2b) is an important cytokine used for antiviral and anticancer therapy. Interferons are considered as a primary line of defence for the host immune system against infectious agents and tumor progression [1]. IFNα-2b is on the World Health Organization’s list of essential medicines. Thus, low cost production of IFNα-2b is necessary to provide patients with affordable therapy. It is approved around the world for the treatment of various diseases including chronic hepatitis C, chronic hepatitis B, hairy cell leukemia, chronic myelogenous leukemia, multiple myeloma, follicular lymphoma, carcinoid tumor, and malignant melanoma. Several host systems have been used for production of IFNα-2b including *Escherichia coli* [2], *Saccharomyces cerevisiae* [3], *Bacillus subtilis* [4], *Pichia pastoris* [5, 6], *Lactococcus lactis* [7], *Yarrowia lipolytica* [8], and mammalian cells [9].

The yield of recombinant interferon from *E.coli* is by far higher than the other systems reported, but there are several drawbacks. The interferon expressed in *E.coli* often forms insoluble, misfolded inclusion bodies that need solublization and refolding steps that could affect the integrity of the refolded proteins [10–12]. The best yield of IFNα-2b after refolding and purification was reported to be 3 g/L from *E. coli* [13]. Avoiding these disadvantages, IFNα-2b has been successfully expressed in several secreted systems. The maximum expression in *Pichia pastoris* has been reported to be around 600 mg/L [6].

The filamentous fungus *Trichoderma reesei* is one of the main producers of lignocellulose degrading enzymes used by enzyme industries world-wide. It is suitable for large scale fermentation processes and has a long history of safe use in the enzyme production industry. Several enzymes produced by *T. reesei* have obtained the generally recognized as safe (GRAS) status by the U.S. Food and Drug Administration. The highest published amount of extracellular protein produced by *T. reesei* was over 100 g per liter [14], thus it has tremendous prospects to produce large amounts of therapeutic proteins based upon its excellent secretion abilities. Furthermore, *T. reesei* is a low cost production system that can be cultivated on inexpensive medium with relatively short cultivation times.

Production of fungal proteases has long been identified as a significant barrier to achieving high production levels of heterologous proteins [15, 16]. In microbial production systems the protease problem has been reduced or overcome by deleting multiple protease genes [17–21]. We have been developing *T. reesei* for use as a therapeutic protein production host with particular focus on reducing the secreted protease activity. We have previously reported identifying 13 major protease enzymes and making deletion strains to reduce the total secreted protease activity [22]. In this earlier work we have deleted seven of the most problematic proteases consecutively from the same strain. In the current report we have improved the previously reported protease deletion strain by first removing the *pep5* aspartic protease and then constructed an IFNα-2b production strain. From this production strain we made a series of protease gene deletions to find out which deletions were most beneficial to the IFNα-2b production level. This is the first study to report interferon production in *T. reesei*.

## Methods

### Gene numbers in this study

The following *T.reesei* genes were referred to in this study: *pep2* (tre53961), *pep5* (tre81004), *pep8* (tre122076), *pep9* (tre79807), *pep11* (tre121306), *pep12* (tre119876), *slp2* (tre123244), *slp7* (tre123865), *slp8* (tre58698), *sep1* (tre124051), *amp1* (tre81070), *amp2* (tre108592), *mep1* (tre122703), *cbh1* (tre123989), *pyr4* (tre74020), *kex2* (tre123561), and *cdna1* (tre110879). The gene identifiers are listed according to the Joint Genome Institute *T. reesei* assembly release version 2.0.

### Creation of *pep5* deletion constructs

A deletion vector was created for the *pep5* aspartic protease gene tre81004. The deletion vector contained the 5′ and 3′ flanking regions of *pep5*, a selection marker with a loopout fragment, and the pRS426 vector backbone. The 5′ flanking region of 1348 bp, the 3′ flanking region of 1164 bp, a 300 bp stretch from the end of *pep5* 5′ flank, and the double selection marker, *pyr4*-*bar*, were amplified by PCR (Additional file 1: Table S1). Template DNA used to amplify these fragments was from the *T. reesei* wild type strain QM6a, which is the genome sequenced strain. PCR amplification was performed with phusion polymerase (Thermo Scientific) with HF buffer. To prepare the vector backbone pRS426 for cloning, it was digested with restriction enzymes EcoRI and XhoI. All PCR reactions and digestion reactions were separated with agarose gel electrophoresis and DNA isolated with a gel extraction kit (Qiagen).

The purified DNA fragments were transformed into *S. cerevisiae* (strain H3488/FY834) to create the final deletion vector, pTTv202. This homologous recombination based cloning method facilitates vector creation as described in Gietz et al. [23]. All DNA fragments to be combined contained 40 base pair overlapping sequences needed for homologous recombination in yeast. The fully assembled plasmid was recovered from yeast, transformed into *E. coli*, purified, and checked by restriction digests and sequencing.

A second deletion plasmid for the aspartic protease *pep5* (tre81004), pTTv229, was constructed using the plasmid pTTv202 (Additional file 2: Figure S1). The *pyr4*-*bar* double marker was removed from pTTv202 with NotI digestion and replaced with a *pyr4* loopout marker. The *pyr4* marker gene was isolated from an existing plasmid after NotI digestion. The new marker was added to pTTv202 with standard ligation using T4 DNA ligase at room temperature. The ligation mixture was transformed into *E. coli*, purified, and verified by restriction digests and sequencing.

### Generation of the 8-protease deletion strain

We have previously reported the generation of a 7-protease deletion strain [22]. In the *pep3* protease locus (tre121133), we added an expression cassette for the native *kex2* gene (tre123561) under control of the *cdna1* promoter (tre110879). The vector contained 5′ and 3′ flank sequences of *pep3*, *cdna1* promoter, the native *kex2* coding sequence, *trpC* terminator, *pyr4* expression cassette, and 300 bp of *pep3* 3′ flank direct repeat fragment. These fragments were amplified by PCR (Additional file 1: Table S2) and assembled into the pRS426 vector backbone by the yeast recombination method [23]. The *kex2* overexpression vector was named pTTv205 (Additional file 3: Figure S2).

To release the *kex2* expression cassette, the plasmid pTTv205 was digested with PmeI + SbfI and the correct fragment was purified from an agarose gel using a QIAquick Gel Extraction Kit (Qiagen). Approximately 5 μg of the expression cassette was used to transform the strain M496 (*pyr4*-). Preparation of protoplasts, transformation, and PCR screening were carried out essentially as described previously [24]. Primers for PCR screening are listed (Additional file 1: Table S2) using Phire Plant Direct kit (Finnzymes, F-130). Correct clones were purified and stored as spore stocks at −80 °C. The final clones were verified by Southern blots.

We continued with this strain and further deleted an additional aspartic protease *pep5* (tre81004). To generate a marker-free protease deletion strain, the *pyr4* marker was removed from the strain as described previously [22]. Consecutive 5-FOA selection steps were carried out to ensure that the clones selected were originating from single cells. Final clones were verified by PCR (Additional file 1: Table S3). Signal corresponding to successful removal of the deletion cassette was obtained for majority of the clones. Removal of the deletion cassette was further verified by plating the clones onto minimal medium plates with or without 5 mM uridine. No growth was observed on the plates without uridine supplementation. Southern analyses verified the removal of the deletion cassette marker.

To release the *pep5* deletion cassette, the plasmid pTTv229 was digested with PmeI + SbfI and the correct fragment was purified from an agarose gel using a QIAquick Gel Extraction Kit (Qiagen). Approximately 5 μg of the deletion cassette was used to transform the deletion strain M496 (with *kex2* overexpression, *pyr4*-). Preparation of protoplasts and transformation were carried out essentially as described previously [24].

Transformants were picked and streaked onto minimal media plates with 1 ml/L triton-X100. The transformant streaks were screened by PCR (Additional file 1: Table S3) to check for proper locus integration of the deletion construct and absence of the protease gene open reading frame. Transformants that were positive for protease gene deletion were purified to single spore clones. The *pep5* gene deletion was verified by Southern analyses from DNA extracted with an Easy-DNA kit (invitrogen) and radiolabeled (^32^P), using the HexaLabel Plus kit (fermentas). Southern digestion schemes were designed using Geneious Pro 5.3.6 software. Southern analyses also verified that the transformant contained only one integrated copy of the deletion construct. The final 8-protease deletion strain was named M504.

### Bioreactor cultivation of the 8-protease deletion strain

The M504 strain was cultivated in a bioreactor to observe the growth characteristics of the strain and obtain culture supernatant for protease purification. Inoculum was cultivated in shake flasks for 2 days at 28 °C, shaking at 200 rpm in medium containing 15 g/L whole spent grain, 30 g/L glucose, 5 g/L (NH_4_)_2_SO_4_, 15 g/L KH_2_PO_4_ at pH 4.5. 100 mL of preculture inoculum was added to 900 mL culture medium to begin the cultivations in 1L bioreactors (Sartorius Biostat Q Plus). The M504 strain was cultured in 30 g/L glucose, 60 g/L lactose, 60 g/L whole spent grain, 5 g/L (NH_4_)_2_SO_4_, 5 g/L KH_2_PO_4_, with the temperature shifting from 28 to 22 °C at 48 h after the exhaustion of glucose. The pH was kept at pH 4.5 with addition of 5 % NH_4_OH and 15 % H_3_PO_4_. The dissolved oxygen saturation level was greater than 30 %. The agitation rate was set between 500 and 1200 rpm with a tip speed of 1.1–2.7 m/s. The total air flow was kept constant at 0.75 L/min. Manual antifoam control was done using dow corning 1500.

### Protease purification

The proteases from the M504 strain bioreactor cultivation samples from day 4 and 5 were purified with pepstatin A and SBTI affinity columns. Aspartic proteases were affinity purified from supernatant using pepstatin A attached to agarose (Sigma #P2032). The supernatant (15 ml) was batch bound to the resin in 35 ml buffer containing 50 mM sodium acetate and 0.2 M NaCl at pH 3.0. The column was washed with the same binding buffer and bound protein was removed with elution buffer (50 mM Tris–HCl, 1 M NaCl, pH 8.5). Fractions of 0.5 ml were collected and subjected to a protein quantitation assay using BioRad Bradford reagent with bovine immunoglobulin as a standard. The protease activity was measured from the collected fractions with a casein substrate to identify the peak fraction.

A 20 ml sample of M504 culture supernatant from day 4 and 5 was incubated with the SBTI-agarose affinity resin (Sigma #T0637; 1 ml) in 30 ml of binding buffer (50 mM Tris, 0.5 M NaCl, pH 7.5). The supernatant binding buffer mixture was added to a 50 ml conical tube and agitated at room temperature for 1 h. The mixture was then poured into a glass column and washed with 200 ml of binding buffer. 50 ml of high salt buffer (1 M NaCl) was next used to further remove nonspecific interactions. Finally, the column was washed again with 100 ml of the original binding/wash buffer. The column was then eluted with 0.8 M benzamidine HCl in 50 mM Tris, pH 5.0. The fractions were collected in 0.5 ml volumes and subjected to a protein quantitation assay using BioRad Bradford reagent with bovine immunoglobulin as a standard. The peak fraction was washed in a Vivaspin ultrafiltration spin filter (Sartorius-stedim) with 10 kDa molecular weight cut-off to remove the benzamidine inhibitor and concentrate the fraction. The protease activity of the collected fractions was measured with a fluorescent casein substrate to identify the peak fraction.

The purified fractions were subjected to trypsin digestion with sequencing grade modified trypsin (Promega #V5111). The resulting peptides were purified by C_18_ ZipTip (Millipore #ZTC18M096). The purified peptides were analyzed by LC–MS/MS on a QSTAR Pulsar, ESI-hybrid quadrupole-TOF (AB Sciex) at the Turku Biotechnology Centre Proteomics Facility.

### Total protease activity assays with casein

Protease activity against casein was tested using the EnzChek protease assay kit (Molecular probes #E6638, green fluorescent casein substrate). The working solution was prepared by diluting the stock to 10 µg/ml in 50 mM sodium citrate, pH 4.5. The purified protease fractions were diluted with sodium citrate buffer. 100 µl of the diluted substrate was combined with the diluted protease fractions in a 96 well sample plate. The plate was then covered and kept at 37 °C for 1–3 h. Fluorescence readings were taken at 1, 2, and 3 h with a Varioskan fluorescent plate reader (Thermo Scientific) using 485 nm excitation and 530 nm emission.

### Generation of the IFNα-2b production strain M577

To construct the IFNα-2b expression vector, a gene encoding IFNα-2b was codon optimized for *T. reesei* expression and synthesized along with the CBHI carrier by Geneart. The IFNα-2b was expressed as a CBHI carrier protein fusion with a KEX2 protease cleavage site, NVISKR, between the carrier and IFNα-2b. A modified version of the CBHI carrier was used to construct the expression cassette where the proline-glycine-proline (PGP) sequence was deleted from the C-terminus of the CBHI carrier amino acid sequence. The PGP sequence possibly introduces a rigid structure next to the KEX2 cleavage site and potentially decreases the KEX2 cleavage efficiency.

**Fig. 1:**
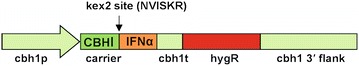
The design of the IFNα-2b expression construct. IFNα-2b expression is driven by the *cbh1* promoter (p) and terminated with the *cbh1* terminator (t). IFNα-2b is expressed as a cleavable fusion protein with CBHI. The NVISKR sequence between CBHI and IFNα-2b can be intracellularly cleaved by the KEX2 protease, which resides in the late golgi. The construct contains a hygromycin selection marker (hygR). The construct is targeted to the *cbh1* locus via the *cbh1*p on the 5′ end and *cbh1* 3′ flank sequence

The expression vector pTTv173 (IFNα-2b) was assembled with the yeast recombination cloning method. The fragments for cloning were cut from the Geneart plasmids with restriction enzymes and were inserted into PacI linearized pPU1-1 vector backbone. Figure 1 shows the design of the expression construct. The expression vector contained targeting sequence for the *cbh1* locus and the hygromycin selection marker. After plasmid rescue and transformation into *E. coli*, all constructs were verified by sequencing. The pTTv173 originally carried the hygromycin marker, but it was changed to acetamide selection. The pTTv173 was digested with NotI and the hygromycin marker was removed. A NotI fragment containing an acetamide selection marker was ligated into the vector backbone to create the pTTv254 expression plasmid. The expression cassette was liberated from the plasmid with PmeI restriction enzyme and purified from an agarose gel.

To generate the IFNα-2b producing strain, the M504 strain was transformed with the IFNα-2b expression cassette (pTTv254) using acetamide for selection [25]. Transformants were screened first by PCR for 5′ and 3′ flank integration and absence of the open reading frame (Additional file 1: Table S4). Southern blot analysis was done to confirm the integration of the IFNα-2b expression construct into the *cbh1* locus. The probes used in the Southern experiments are listed in Additional file 1: Table S5. The final IFNα-2b production strain with 8 protease deletions was named M577.

### Generation of 9-protease deletion strains from M577

Deletion constructs for the *slp7*, *pep8*, *pep9*, *pep11*, *mep1*, *amp1*, *amp2*, and *sep1* were created by PCR and yeast recombination as generally described above for *pep5*. These deletion vectors contained the *pyr4*-hygromycin double marker cassette. The primers for making the constructs are listed in Additional file 1: Table S6–S21.

The deletion constructs were separated from the plasmids via PmeI digestion and gel purified. Protoplasts of strain M577 were transformed with 5 µg of the deletion construct DNA and plated on hygromycin selection plates. Streaks of the transformants were made and screened by PCR for 5′ and 3′ flank integration and absence of the open reading frame. The screening primers are listed in Additional file 1: Table S6-S21. Positive transformants were put through single cell purification and screened a final time by PCR. The final spore suspensions were made and the protease deletion strains given strain numbers. The *slp7* (M673), *pep8* (M670), *pep9* (M671), *pep11* (M672), *amp1* (M669), *amp2* (M674), and *sep1* (M668) deletion strains were completed. The *mep1* deletion strain from vector pTTv468 was not clean so we did not continue with it.

### 9-protease deletion strains producing IFNα-2b in 24 well culture

The protease deletion strain transformants were grown in 24 well culture in TrMM plus 40 g/L lactose, 20 g/L spent grain extract, 8.6 g/L diammonium citrate, 5.4 g/L NaSO_4_, 100 mM PIPPS at pH 4.5, shaking at 28 °C at 800 rpm (Infors AG). TrMM contains 7.6 g/L (NH_4_)_2_SO_4_, 15.0 g/L KH_2_PO_4_, 2.4 mM MgSO_4_-7H_2_O, 4.1 mM CaCl_2_-H_2_O, 3.7 mg/L CoCl_2_, 5 mg/L FeSO_4_-7H_2_O, 1.4 mg/L ZnSO_4_-7H_2_O and 1.6 mg/L MnSO_4_-7H_2_O [24]. Immunoblotting was done to detect IFNα-2b. The supernatant was diluted with water and loading buffer, so that 0.5 µl of each supernatant could be loaded into the 4–20 % Criterion gel (BioRad). Sample was mixed with Laemmli sample buffer containing β-mercaptoethanol and heated at 95 °C for 5 min. The proteins were transferred to nitrocellulose with the Turbo semi-dry blotter (BioRad) for 7 min. The nitrocellulose membrane was blocked with 5 % milk in TBST for 1 h. The IFNα-2b was detected with a mouse anti-IFNα-2b antibody (Abcam #ab9386) diluted 0.5 µg/ml in TBST. The primary antibody was incubated with the membrane for 1 h shaking at room temperature and then the membrane washed briefly with TBST. The secondary antibody was goat anti-mouse AP conjugate (BioRad #170–6520) diluted 1:10,000 in TBST. The secondary antibody was incubated for 1 h shaking at room temperature, the antibody was removed, and membrane washed for 1 h shaking at room temperature. The blot was developed using AP substrate BCIP/NBT (Promega #S3771).

### Bioreactor cultures of the 9-protease deletion strains producing IFNα-2b

Inoculums were cultivated in shake flasks for 2 days at 28 °C, shaking at 200 rpm in medium containing 10 g/L yeast extract and 40 g/L glucose at pH 5.0. 100 mL of preculture inoculum was added to 900 mL culture medium to begin the cultivations in 1L bioreactors (Sartorius Biostat Q Plus). The IFNα-2b production strains were cultured in 40 g/L lactose, 20 g/L spent grain extract, 20 g/L whole spent grain, 5 g/L (NH_4_)_2_SO_4_, 5 g/L KH_2_PO_4_, with the temperature kept constant at 28 °C. The pH was maintained at pH 4.5 with addition of 5 % NH_4_OH and 15 % H_3_PO_4_. The dissolved oxygen saturation level was greater than 30 %. The agitation rate was set between 500–1200 rpm with a tip speed of 1.1–2.7 m/s. The total air flow was kept constant at 0.5 L/min. Manual antifoam control was done using dow corning 1500. These cultures were named Triab 116 (M669), 117 (M670), 118 (M672), 119 (M673), and 121 (M674). Base consumption and CO_2_ generation were measured from these reactors. Supernatant samples were taken regularly for measurement of the IFNα-2b concentration.

A second set of batch cultivations was done with the IFNα-2b expression strains in different medium. The deletion strains were cultivated in 1L batch cultivations using a 2L DASGIP Parallel Bioreactor System. Cultivations have been carried out in a cellulase-inducing medium containing 20 g/L yeast extract, 40 g/L cellulose, 80 g/L cellobiose, and 40 g/L sorbose at pH 4.5 with the temperature shifted from 28 to 22 °C at 48 h. The biomass capacitance in all reactors was monitored on-line using ABER Futura probes (Aber Instruments, Wales, UK). Base consumption and CO_2_ generation were also monitored as indirect measures of biomass. These cultivations were assigned the culture codes FTR108_R1 (M668), FTR108_R4 (M671), FTR108_R5 (M672), FTR108_R6 (M673), FTR108_R7 (M674) and FTR109_R9 (M577). Supernatant samples were regularly taken so that the IFNα-2b concentrations could be measured. The supernatant was diluted in water and loading buffer so that 0.1 µl could be loaded per well. The immunoblotting procedure to detect IFNα-2b was carried out as described above with the 24 well cultures.

The strain M674 (Δ*amp2*) was also cultivated with and without SBTI inhibitor addition. The inoculum was cultivated in a shake flask for 2 days at 28 °C, shaking at 200 rpm in medium containing 10 g/L yeast extract and 40 g/L glucose at pH 5.0. 100 mL of preculture inoculum was added to 900 mL culture medium to begin the cultivations in 1L bioreactors (Sartorius Biostat Q Plus). The growth medium contained 20 g/L yeast extract, 40 g/L cellulose, 80 g/L cellobiose, and 40 g/L sorbose, 5 g/L (NH_4_)_2_SO_4_, 5 g/L KH_2_PO_4_ with the temperature shifting from 28 °C to 22 °C at 48 h. The pH was maintained at pH 4.5 with addition of 5 % NH_4_OH and 15 % H_3_PO_4_. The dissolved oxygen saturation level was greater than 30 %. The agitation rate was set between 500–1200 rpm with a tip speed of 1.1–2.7 m/s. The total air flow was kept constant at 0.5 L/min. Manual antifoam control using PPG2000. The Triab 125 cultivation was done without SBTI inhibitor and Triab 126 was done with SBTI inhibitor feeding to provide a concentration of 0.4 mg/ml.

The culture supernatant was diluted in water so that 0.025 µl could be loaded in 10 µl volume into a 4–20 % Criterion SDS-PAGE gel (Bio-Rad). Immunodetection was done with Abcam (#ab9386) anti-IFNα-2b antibody diluted to 1 µg/ml in TBST. The secondary antibody from Bio-Rad (#170-6520) goat anti-mouse IgG AP conjugated secondary antibody diluted 1:5000 in TBST. The protein standards were loaded in the gel corresponding to 200, 100 and 50 ng of full length IFNα-2b (Acris Antibodies #AR09043PU-N).

### Inhibitor studies with the best IFNα-2b production strains

Cultures were grown in 24 well plates in 3 ml of TrMM plus 40 g/L lactose, 20 g/L spent grain extract, 8.6 g/L diammonium citrate, 5.4 g/L NaSO_4_, with 100 mM PIPPS, pH 4.5 at 28 °C. The 24 well plates were grown shaking at 800 rpm at 28 °C (Infors AG). Inhibitors were added daily from day 3 until day 6. The pepstatin, chymostatin, and bestatin inhibitors were made as 20 mM stocks in DMSO, while the SBTI was made up to 10 mg/ml in Tris buffer pH 7.4. The final inhibitor concentrations were 10 µM pepstatin, 20 µM chymostatin, 0.4 mg/ml SBTI, and 20 µM bestatin. Supernatant samples were taken daily and diluted in water and loading buffer so that 0.2 µl could be loaded per well. The IFNα-2b production level was measured via immunoblotting against an IFNα-2b standard curve.

### IFNα ELISA assay

The culture supernatant containing secreted IFNα-2b was evaluated with the IFN-alpha Multisubtype Human ELISA Kit (Life technologies #KHC4032), according to the manufacturer’s instructions.

## Results

### Protease purification

Our efforts to adapt *T. reesei* for therapeutic protein production led us to successively delete eight protease genes from this host. In order to examine the remaining protease species, the strain M504 was cultivated in the bioreactor at pH 4.5 to assess its growth and to purify aspartic and serine proteases from the supernatant. The proteases binding to pepstatin A and SBTI were purified from day 4 and 5 supernatant samples. The pepstatin A column was able to capture several aspartic proteases and after LC–MS/MS analysis of tryptic peptides these were determined to include PEP2 (12 % sequence coverage), PEP8 (12 % sequence coverage), PEP9 (6 % sequence coverage), PEP11 (1 % sequence coverage), and PEP12 (7 % sequence coverage). The PEP2 protease was the last of the originally identified aspartic proteases that represent the freely secreted aspartic proteases [9], while PEP8, PEP9, PEP11, and PEP12 are predicted to be cell-bound. The other major secreted aspartic proteases have been deleted from the strain. The SBTI inhibitor protein bound to the subtilisin proteins and after LC–MS/MS analysis of tryptic peptides SLP2 (17 % sequence coverage) and SLP8 (1 % sequence coverage). The purification peak fractions from day 5 samples were several times more proteolytically active against the casein substrate compared to values measured on day 4 (Additional file 4: Figure S3). This suggests that there would be more aspartic and subtilisin proteases in the culture medium on day 5 compared to day 4.

### IFNα-2b production strains

The M504 strain was transformed with an IFNα-2b expression construct (Fig. 1) to test its production capability. The resulting M577 strain was checked by Southern analysis and it was confirmed that it contained multiple copies of the IFNα-2b expression cassette. There was one in the *cbh1* locus, but there were one or more copies elsewhere in the genome. Protease deletion strains derived from M577 were completed for Δ*pep8* (M670), Δ*pep9* (M671), Δ*pep11* (M672), Δ*slp7* (M673), Δ*amp1* (M669), Δ*amp2* (M674), and Δ*sep1* (M668) protease genes (Table 1).

**Table 1:** Strains created and used in the study

Strain #	Proteases deleted	# of proteases	Protein expressed
M486^a^	Δ*pep1*Δ*tsp1*Δ*slp1*Δ*gap1*Δ*gap2*Δ*pep4*Δ*pep3*	7	–
M496	Δ*pep1*Δ*tsp1*Δ*slp1*Δ*gap1*Δ*gap2*Δ*pep4*Δ*pep3*, *pyr4*-	7	–
M504	Δ*pep1*Δ*tsp1*Δ*slp1*Δ*gap1*Δ*gap2*Δ*pep4*Δ*pep3*Δ*pep5*	8	–
M577	Δ*pep1*Δ*tsp1*Δ*slp1*Δ*gap1*Δ*gap2*Δ*pep4*Δ*pep3*Δ*pep5*	8	IFNα-2b
M668	Δ*pep1*Δ*tsp1*Δ*slp1*Δ*gap1*Δ*gap2*Δ*pep4*Δ*pep3*Δ*pep5*Δ*sep1*	9	IFNα-2b
M669	Δ*pep1*Δ*tsp1*Δ*slp1*Δ*gap1*Δ*gap2*Δ*pep4*Δ*pep3*Δ*pep5*Δ*amp1*	9	IFNα-2b
M670	Δ*pep1*Δ*tsp1*Δ*slp1*Δ*gap1*Δ*gap2*Δ*pep4*Δ*pep3*Δ*pep5*Δ*pep8*	9	IFNα-2b
M671	Δ*pep1*Δ*tsp1*Δ*slp1*Δ*gap1*Δ*gap2*Δ*pep4*Δ*pep3*Δ*pep5*Δ*pep9*	9	IFNα-2b
M672	Δ*pep1*Δ*tsp1*Δ*slp1*Δ*gap1*Δ*gap2*Δ*pep4*Δ*pep3*Δ*pep5*Δ*pep11*	9	IFNα-2b
M673	Δ*pep1*Δ*tsp1*Δ*slp1*Δ*gap1*Δ*gap2*Δ*pep4*Δ*pep3*Δ*pep5*Δ*slp7*	9	IFNα-2b
M674	Δ*pep1*Δ*tsp1*Δ*slp1*Δ*gap1*Δ*gap2*Δ*pep4*Δ*pep3*Δ*pep5*Δ*amp2*	9	IFNα-2b

^a^M486 was created in the previously reported work [22]

## 24 well cultures of IFNα-2b production strains

**Fig. 2:**
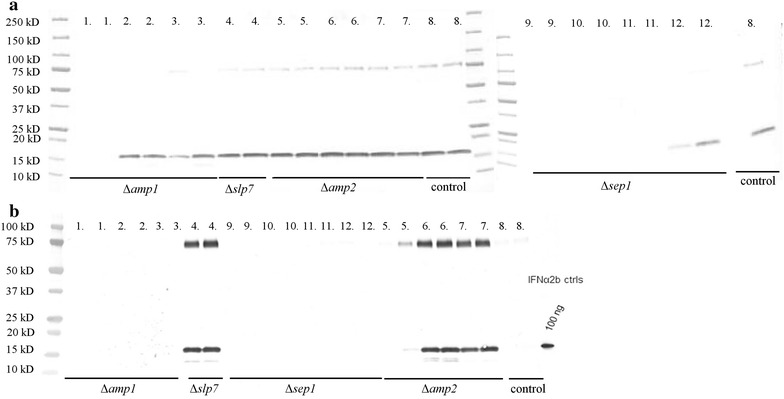
Immunoblot detecting IFNα-2b produced by 9-protease deletion strains. Transformants from *amp1*(#1–3), *slp7* (4), *amp2* (#5–7), and *sep1* (#9–12) deletion strains were grown in duplicate in 24 well plates in TrMM with 8.6 g/L diammonium citrate, 5.4 g/L NaSO_4_, 100 mM PIPPS, 20 g/L spent grain extract, 40 g/L lactose at pH 4.5, shaking at 28 °C. **a** The supernatant from day 6 was diluted so that 0.5 µl could be loaded per well. The M577 strain is the parental control (#8). The full length IFNα-2b runs around 17 kDa and the carrier bound material runs at 75 kDa. **b** Supernatant from day 7 was diluted so that 0.5 µl could be loaded per well. The M577 strain is the parental control (#8)

**Fig. 3:**
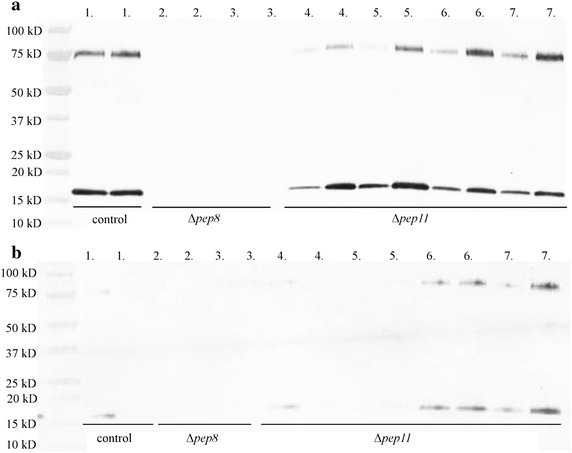
Immunoblot detecting IFNα-2b produced by 9-protease deletion strains. Transformants from *pep8* (#2–3) and *pep11* (#4–7) deletion strains were grown in duplicate using 24 well plates in TrMM with 8.6 g/L diammonium citrate, 5.4 g/L NaSO_4_, 100 mM PIPPS, 20 g/L spent grain extract, 40 g/L lactose at pH 4.5, shaking at 28 °C. M577 is the parental control (#1). **a** The supernatant from day 5 was diluted so that 0.5 µl could be loaded per well. The full length IFNα-2b runs around 17 kDa and the carrier bound material runs at 75 kDa. **b** The supernatant from day 7 was diluted so that 0.5 µl could be loaded per well

The IFNα-2b production strain M577 and the series of 9-protease deletion transformants were first cultured in 24 well plates to select transformants and to generally assess the productivity of the different deletions. The M671 strain was not ready at this stage. The best transformants from M668, M669, M672, and M673, and M674 produced IFNα-2b at similar levels on day 6 (Figs. 2a, 3a), but on day 7 there was a clear difference for the M673 and M674 transformants (Figs. 2b, 3b). The M673 and M674 strains allowed for stable expression up to day 7, whereas the other strains and M577 showed a very low level or no IFNα-2b. There was free IFNα-2b at 17 kDa, but there was also a CBHI carrier bound form at 75 kDa. The KEX2 cleavage site between the IFNα-2b and the CBHI carrier was not fully processed in this case under these conditions.

The pH of the culture also had an influence on how effective the deletions appeared to be. For example when we cultured these strains at pH 5.5, instead of pH 4.5, the M668 (Δ*sep1*) strain gave the biggest improvement (Additional file 5: Figure S4). The M673 and M674 strains also showed a positive effect at pH 5.5.

**Fig. 4:**
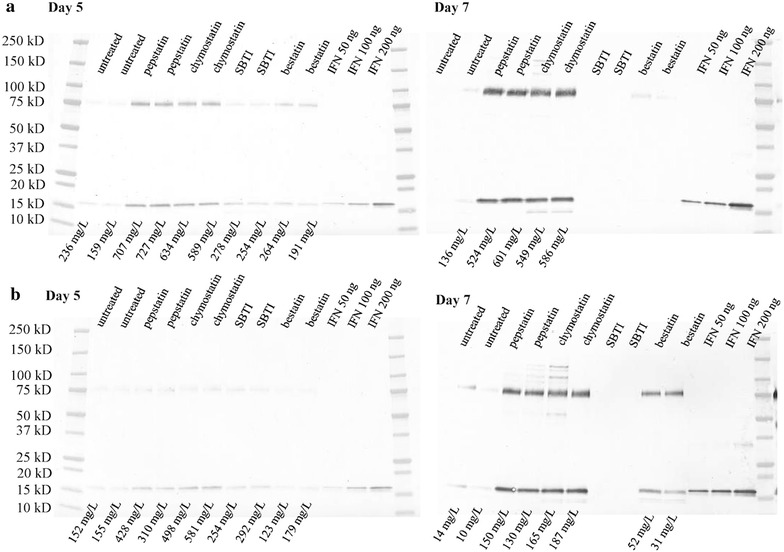
Immunoblot detecting IFNα-2b production. **a** M673 (Δ*slp7*) strain treated with protease inhibitors. Cultures were grown in 24 well plates in TrMM plus 20 g/L spent grain extract and 40 g/L lactose, with 8.6 g/L diammonium citrate, 5.4 g/L NaSO_4_, 100 mM PIPPS, pH 4.5 at 28 °C. Duplicate wells were used per treatment. The day 5 and day 7 supernatants were diluted so that 0.2 µl could be loaded per well. 10 µM pepstatin, 20 µM chymostatin, 0.4 mg/ml SBTI, and 20 µM bestatin were used as protease inhibitors. **b** M674 (Δ*amp2*) strain treated with protease inhibitors. The day 5 and day 7 supernatants were diluted so that 0.2 µl could be loaded per well

The two best deletion strains were grown in 24 well plates along with protease inhibitors to check how the expression could be further improved and to learn what types of proteases were limiting stability. The M673 (Δ*slp7*) strain produced up to 236 mg/L of full length IFNα-2b on day 5 (Fig. 4a). The addition of pepstatin A increased the production level to 727 mg/L and chymostatin improved the level to 634 mg/L. The SBTI and bestatin treatments did not result in noticeable improvement under these conditions. On day 7, the pepstatin and chymostatin treated cultures continued to produce high levels of IFNα-2b, while the level was very low or below detection in the other cultures. The inhibition of aspartic proteases and subtilisins provided a major benefit for IFNα-2b stability. On day 7 there was a lower molecular weight degradation product below the main band in the chymotrypsin treated lanes, but not in the pepstatin treatments. This suggests that aspartic proteases are responsible for creating this degradation product. The production levels with both treatments were 524–601 mg/L.

Similar observations were made with the M674 (Δ*amp2*) strain. On day 5 the expression levels reached 155 mg/L, while pepstatin treatment pushed this up to 428 mg/L and chymostatin allowed up to 581 mg/L of IFNα-2b production (Fig. 4b). SBTI provided protease reduction and facilitated up to 292 mg/L of IFNα-2b. The metalloprotease inhibitor selective for aminopeptidases, bestatin, provided a slight benefit up to 179 mg/L. The scene changed dramatically by day 7. The untreated cultures reduced to 14 mg/L, but the pepstatin and chymostatin maintained levels between 130 and 187 mg/L. The chymostatin treatment was maybe slightly more effective. The aminopeptidase inhibitor was effective at improving the production up to 52 mg/L. The SBTI inhibitor did not show any major benefit on this day. SBTI loses its effectiveness as the pH becomes more acidic. It has optimal activity around neutral pH, but in more acidic cultures the inhibition activity is minimal. The starting pH of these cultures was pH 4.5, but reduced to pH 4.0 around day 7.

On both days and with both strains there was a significant amount of CBHI carrier bound IFNα-2b present at 75 kDa. There was generally an equal or greater amount of IFNα-2b bound to the carrier compared to what was in free form. These studies demonstrate the overall expression potential of these strains. If a few more proteases were deleted we could achieve these production levels without protease inhibitors.

### Bioreactor cultivations

The best transformants from each deletion were first cultured in 40 g/L lactose, 20 g/L spent grain extract, and 20 g/L whole spent grain containing medium in cultivations Triab 116, 117, 118, 119 and 121. The highest detectable product level that M577 achieved under these conditions and in this medium was 0.50 g/L on day 3. With the M673 (Δ*slp7*) strain 0.94 g/L of product was measured on day 3 and M674 (Δ*amp2*) achieved 1.14 g/L on day 3, which was the time-point of the highest IFNα-2b concentration in these batch cultivations (Table 2) (Additional file 6: Figure S5). On day 4 these cultures are already reducing in expression and on day 5 the cultures are complete. We have observed for M504 that on day 5 there was several fold more protease activity from purified protease fractions compared to day 4 (Additional file 4: Figure S3). Thus, the complete degradation of IFNα-2b on day 5 is probably due to increased protease activity. On day 3 with the M669, M670, and M672 strains less product was detected than with M577 strain under the same conditions.

**Table 2:** Bioreactor base consumption data (ml) and IFNα-2b expression level data (g/L)

Strain #	Deletion	Base consumption (ml)					IFNα-2b level (g/L)		
		Day 1	Day 2	Day 3	Day 4	Day 5	Day 2	Day 3	Day 4
M577	Parental	3	8	12	24	40	0.2	0.5	0.1
M669	Δ*amp1*	2	10	21	56	58	0.2	0.2	0.1
M670	Δ*pep8*	5	10	10	10	11	0.2	0.0	0.0
M672	*Δpep11*	4	8	11	23	38	0.3	0.2	0.1
M673	*Δslp7*	3	10	20	44	57	0.3	0.9	0.5
M674	*Δamp2*	3	8	13	31	45	0.6	1.1	0.5

Based upon base consumption measurements, M669 and M673 grew slightly faster than M577, whereas the M670 strain grew slower under these conditions (Table 2). Notably, growth and sporulation were affected by the Δ*pep8* (M670) when growing on plates. Comparing the data in Table 2 there was no correlation between base consumption and production level observed. Base consumption data suggests the highest biomass for the M669 strain, but this did not result in higher production levels of IFNα-2b. The best production strain in this cultivation series M674 consumed nearly the same amount of base on day 3 as compared to M577, yet it produced 2.3 times more IFNα-2b. This is expected to be due to reduced secreted protease activity from M674.

These batch cultivations were grown in 40 g/L lactose, 20 g/L spent grain extract, and 20 g/L whole spent grain at pH 4.5 (Triab 116-119, 121 cultivations). Base consumption was used to monitor the growth of the fungus in the bioreactor and serves as an indirect measure of biomass.

Clearly, the M673 (Δ*slp7*) and the M674 (Δ*amp2*) deletion strains were the best producers of IFNα-2b in the bioreactor and in the 24 well culture studies. The media in both cultures was very similar, but in the fermentation medium we had 20 g/L of solid spent grain to provide better induction and expression potential. The pH in the bioreactor was better controlled keeping it firmly at pH 4.5, whereas the pH in the 24 well cultures became slightly more acidic. Thus, in the bioreactor with better cultivation conditions and media we could achieve several fold higher maximum production levels. The processing of the KEX2 cleavage site between the IFNα-2b and the CBHI carrier was much better under bioreactor conditions compared to what was seen in the 24 well studies. There was very little carrier bound product at 75 kDa in samples from the best deletion strains. In the M674 strain, the carrier bound product was almost not visible (Additional file 6: Figure S5).

We cultured the best strains and additional strains in bioreactors with different culture medium. They were grown in 20 g/L yeast extract, 40 g/L cellulose, 80 g/L cellobiose, and 40 g/L sorbose at pH 4.5. The expression results are shown in Table 3 (Additional file 7: Figure S6). Immunoblotting was used to quantify the expression level of IFNα-2b produced. The parental M577 control cultivation produced 0.8 g/L of detectable IFNα-2b on day 3. The M674 (Δ*amp2*) strain was the most improved strain overall, providing 2.4 g/L of measureable product on day 3. Deletion of M674 (Δ*amp2*) provided a 2.9-fold improvement in IFNα-2b production. The M673 (Δ*slp7*) strain achieved 2.1 g/L on day 4. This was a 2.5-fold improvement over the parental strain M577. The M668 (Δ*sep1*) strain secreted 1.4 g/L of measureable IFNα-2b. The M671 (Δ*pep9*) strain also improved the IFNα-2b expression level to 1.0 g/L. The M672 (Δ*pep11*) strain produced 0.7 g/L under these conditions. Under these conditions there was more IFNα-2b bound to the CBHI carrier, compared to earlier bioreactor cultures described with spent grain and lactose medium. We did not check the biological activity of the secreted IFNα-2b. However, the folding of the IFNα-2b was evaluated using a sandwich ELISA. Two different antibodies were used to capture and detect IFNα-2b. This assay successfully detected IFNα-2b in a natively folded structure and measured 2.4 g/L of IFNα-2b on day 4, which was the same as what was measured by immunoblotting.

**Table 3:** Bioreactor data and IFNα-2b expression level data (g/L)

Strain #	Deletion	Capacitance (pF/cm)					IFNα-2b level (g/L)	
		Day 1	Day 2	Day 3	Day 4	Day 5	Day 3	Day 4
M577	Parental	n/a	n/a	n/a	n/a	n/a	0.8	0.6
M668	Δ*sep1*	5	11.5	19.9	25	21.5	1.3	1.4
M671	Δ*pep9*	5	9.9	15.1	20.5	21.5	0.9	1.0
M672	Δ*pep11*	4.9	6.9	12.2	17.1	20.8	0.7	0.6
M673	Δ*slp7*	2.5	6.8	13.4	19.1	21.2	1.4	2.1
M674	Δ*amp2*	4.9	7.5	13.4	18.1	22.2	2.1	2.4

Strain #	Deletion	Base consumption (ml)					IFNα-2b level (g/L)	
		Day 1	Day 2	Day 3	Day 4	Day 5	Day 3	Day 4
M577	Parental	4.8	17	29	48	63	0.8	0.6
M668	Δ*sep1*	2	17	37	64	68	1.3	1.4
M671	Δ*pep9*	1	14	27	47	65	0.9	1.0
M672	Δ*pep11*	0	9	20	38	63	0.7	0.6
M673	Δ*slp7*	0	8	30	53	76	1.4	2.1
M674	Δ*amp2*	0	8	27	52	70	2.1	2.4

These batch cultivations were grown in 20 g/L yeast extract, 40 g/L cellulose, 80 g/L cellobiose, and 40 g/L sorbose at pH 4.5. Capacitance (pF/cm) and base consumption (ml) data were used to monitor the growth of the fungus in the bioreactor. These both serve as indirect measures of biomass.

From these cultivations we measured capacitance, base consumption, and CO_2_ as indicators of fungal growth. Based on these measurements, the growth of strains M671 (Δ*pep9*), M673 (Δ*slp7*) and M674 (Δ*amp2*) was comparable to M577 (Table 3, Additional file 8: Figure S7) indicating comparable biomass levels at day 3 and 4 with peak cell density at day 5. The M672 (Δ*pep11*) strain was slightly slower growing, whereas the M668 (Δ*sep1*) strain grew slightly faster than M577, showing peak cell density already on day 4 (Table 3, Additional file 8: Figure S7). On day 5 the capacitance and base consumption readings are generally very similar. Interestingly, peak IFNα-2b production levels were observed on day 3 or 4 (depending on the strain), but not on day 5 where biomass levels are highest. This suggests that despite the expectation of higher IFNα-2b expression levels on day 5, detectable IFNα-2b levels are likely declining due to increasing protease activity.

As seen in the earlier bioreactor cultivations increased biomass was not a good indicator for the expression level of IFNα-2b. When comparing base consumption data on day 3 the M674 strain used 2 ml less base compared to M577, but produced 2.6 times more IFNα-2b. On day 4, M674 consumed only 4 ml (8 %) more base, but produced 4 times more IFNα-2b. Also, the M673 strain demonstrated the same results in this regard. Even though M668 grew faster and had more biomass on day 3 and 4, the expression levels for M673 and M674 were higher.

The M674 (Δ*amp2*) strain was also cultivated with and without SBTI inhibitor addition in a different set of bioreactors than used above. The medium contained 20 g/L yeast extract, 40 g/L cellulose, 80 g/L cellobiose, and 40 g/L sorbose at pH 4.5 with the temperature shifting from 28 to 22° at 48 h. The Triab 125 cultivation was done without SBTI inhibitor and Triab 126 was done with SBTI inhibitor feeding. The base consumption and CO_2_ measurements indicated that the cultures grew nearly identically. The addition of the SBTI inhibitor did not affect the growth rate of the culture. The rates of cellobiose and sorbose consumption were also similar. The cellobiose concentration was down to 10 g/L by 96 h and completely consumed at 120 h. Sorbose was used at a slower rate and reached 10 g/L at 148 h.

**Fig. 5:**
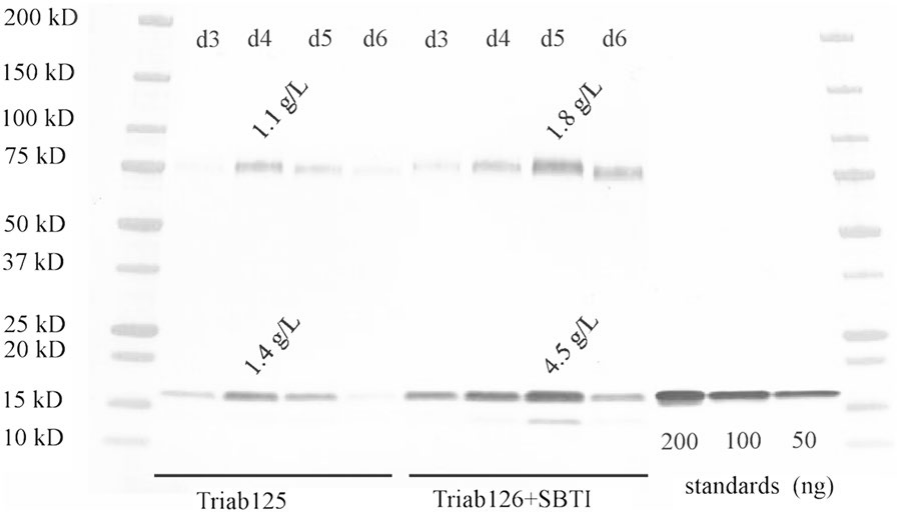
Immunoblot showing the IFNα-2b production level in the M674 supernatant cultivated in Triab125 and Triab126. Bioreactor cultures were grown in 20 g/L yeast extract, 40 g/L cellulose, 80 g/L cellobiose, and 40 g/L sorbose, 5 g/L (NH_4_)_2_SO_4_, 5 g/L KH_2_PO_4_ at pH 4.5. The Triab126 cultivation was done with SBTI inhibitor feeding (0.4 mg/ml final concentration). The supernatants were diluted so that 0.025 µl could be loaded per well. The protein standards were loaded in the gel corresponding to 200 ng, 100 ng, and 50 ng of full length IFNα-2b. The full length IFNα-2b runs around 17 kDa and the carrier bound material runs at 75 kDa

The SBTI inhibitor improved the IFNα-2b expression level by over threefold (Fig. 5). The base level was 1.4 g/L on day 4, but with protease inhibitor treatment the IFNα-2b expression could be increased to 4.5 g/L on day 5. The addition of inhibitor shifted the peak expression day until day 5, which indicated higher stability of the IFNα-2b in the supernatant. The M674 base culture also produced 1.1 g/L of CBHI bound IFNα-2b and 1.8 g/L of bound material when SBTI was added. Adding up the free and bound forms the M674 strain secreted around 2.5 g/L of total IFNα-2b under standard conditions, but when the serine protease activity was reduced by inhibitor the total secretion reached 6.3 g/L.

## Discussion

Work with protease inhibitors and affinity purification has led the way to identifying which proteases present challenges for IFNα-2b stability in *T. reesei* cultures. We identified that pepstatin A, chymostatin, and soybean trypsin inhibitors were the most effective at stabilizing IFNα-2b against proteases. A selection of these inhibitor binding proteases and their homologs were chosen for deletions of the respective genes. Making a deletion strain series such as this was the main strategy driving further strain development. By making a range of protease deletions we could evaluate any potential improvements in production of IFNα-2b as a function of lower protease activity and at the same time evaluate if the deletion led to any potential growth defects or phenotypical changes.

The best production strains we report here have 9 protease gene deletions. The major acidic proteases including *pep1*, *pep3*, *pep4*, *pep5*, *gap1*, and *gap2* and the serine proteases *tsp1* and *slp1* were previously deleted from the strain [22]. Our previous inhibitor studies in the 6-protease deletion background indicated that pepstatin treatment of the supernatant improved the stability of IFNα-2b [22]. Thus, we have aimed to remove all the aspartic protease activity from the supernatant. The original *T.reesei* production strain secreted at least 8 aspartic proteases and at least 2 glutamic proteases. Additionally, there were at least 2 serine proteases remaining in the 6-protease deletion strain that were active under acidic conditions that lead to IFNα-2b instability.

Proteomic studies on *T. reesei* QM6a have identified up to 39 secreted proteases from culture supernatant [26]. It is easy to understand why proteases have been considered a major barrier to producing heterologous proteins, especially for sensitive therapeutic proteins like hormones and cytokines that are by nature unstable. These 39 secreted proteases were identified while studying the secretion of cellulase and hemicellulase enzymes and not in the context of improving heterologous protein expression [26, 27]. In contrast, we have focused on finding those responsible for therapeutic protein degradation. So far we have identified up to 17 proteases that are related to degradation of therapeutic antibodies, IFNα-2b, and insulin-like growth factor [22]. In the current study we have purified the PEP2, PEP8, PEP9, PEP11, PEP12, SLP2, and SLP8 from the M504 strain grown at pH 4.5. The pepstatin and chymostatin inhibitors were very effective at inhibiting degradation in the best IFNα-2b production strains M673 (Δ*slp7*) and M674 (Δ*amp2*). Further progress to increase yields can be made by making consecutive deletions of SLP2, SLP8, and the remaining aspartic proteases.

The *pep8*, *pep9*, *pep11*, and *pep12* aspartic proteases are different than the freely secreted aspartic proteases, since they contain an additional C-terminal domain that could be GPI anchored. These anchors are cut away under certain conditions, because these proteases were also found in the supernatant. Work on yeast aspartic proteases suggests that fungal yapsins are involved in cell-wall assembly and/or remodelling [28]. Additionally, yapsins have been shown to be involved in antibody degradation when antibodies are produced in some yeasts [21]. The yeast yapsins were cutting the antibody in the heavy chain hinge region. Thus, we were interested in investigating *Trichoderma* yapsin-like proteases so that a production system could be developed to produce antibodies as well. It was important to first screen deletions of these yapsin-like aspartic proteases, because unlike the freely secreted aspartic proteases these membrane associated relatives had greater potential to cause changes in strain growth and sporulation, as we have observed under some conditions with the *pep8* deletion. However, in these cases we could simply develop more suitable culture processes or employ a mild gene silencing approach to reduce the expression of a specific protease gene [29].

The best protease gene deletions in this limited series were Δ*slp7* and Δ*amp2*. The SLP7 subtilisin related protease is classified as a sedolisin protease [30]. These are interesting because they are trypsin-like serine proteases with acidic pH optima. Sedolisins, serine-carboxyl peptidases, are proteolytic enzymes whose fold resembles that of subtilisin; however, they are considerably larger. Deletion of the *slp7* protease in this work greatly benefited the production of IFNα-2b at pH 4.5. This protease was identified in *T. reesei* earlier as it binds to the soybean trypsin inhibitor [22]. We deleted it in the present study to check its potential and importance under production conditions. It turned out to be quite influential to improve IFNα-2b expression. There are two other sedolisins expressed in the strain, but sequence homology suggests that they are tripeptidyl peptidase-like proteases and likely do not have endoprotease activity.

The metalloprotease *amp2* is related to several other metalloproteases isolated in our earlier study. The protease domain belongs to the zinc metalloprotease family, M1 subtype (aminopeptidase N) [31]. The aminopeptidase N description suggests that it may be a broad specificity aminopeptidase, but may have some preference for alanine. Yet, as the deletion strain suggests it is definitely not limited to aminopeptidase activity, it appears to have endoprotease capability. Interestingly, this enzyme is unlike all the other proteases we have worked with, because sequence homology suggests it is a bifunctional enzyme that contains a leukotriene A4 hydrolase domain used for lipid metabolism. The leukotriene A4 hydrolases have been characterized in mammals, but little is known about their function in lower organisms [32, 33].

Carrier protein fusions have been used in fungal protein production systems to promote more efficient secretion of a heterologous protein by coupling it to a secreted fungal protein, such as a cellobiohydrolase [34]. An important consideration when using this approach was the cleavage of the CBHI carrier from the IFNα-2b. We have used the KEX2 cleavage site NVISKR between the carrier and the IFNα-2b protein. The NVISKR sequence is derived from the pro-peptide of *Aspergillus niger* glucoamylase and is a target site for a KEX2-type protein processing protease. This cleavage sequence has been used in *Aspergillus* production systems with variable success [35–37]. In designing a fusion strategy we used a modified CBHI carrier that had proline-glycine-proline (PGP) sequence deleted from the C-terminus. It was thought that the PGP sequence may generate a rigid structure next to the KEX2 cleavage site and potentially decrease the KEX2 cleavage efficiency. As can be seen in some of the cultivations, IFNα-2b was not always fully released from the carrier, even though we had overexpressed the *kex2* protease. There are several potential reasons. The strains grow and produce much better on the yeast extract based medium. For example, under those more optimal conditions the high level of expression could have overwhelmed the capacity of KEX2 to process all the fusion protein. In contrast, the carrier cleavage was excellent in the bioreactor cultivations grown in spent grain and lactose, but this may be related to the lower expression levels in that medium. The observed difference in cleavage efficiency may also, to some extent, be explained by cleavage of the fusion protein by supernatant proteases. If the secreted protease activity were higher in the lactose and spent grain medium, this may help explain the media difference. However, we did not compare the protease activity from these two cultivations during this study.

Regardless of the reason, the results demonstrate that, in general, the carrier construct with NVISKR cleavage site works well, but further KEX2 cleavage site optimization can lead to full carrier release and enhanced production of heterologous proteins [38, 39]. The residues around the KEX2 site may also influence the cleavage efficiency. For example, adding three glycine residues after the NVISKR sequence improved the fidelity and efficiency of KEX2 cleavage in *A. niger* [36, 40]. Further work will be done to find alternative carrier constructs and cleavage sites.

## Conclusions

Our system currently produced 2.4 g/L of IFNα-2b and has upward potential up to 4.5 g/L, with protease inhibitor treatment, based upon the current cultivation conditions and medium. These levels already compete well with those obtained in *E. coli* from an inclusion body refolding production process [13] and are by far higher than levels reached in *Pichia pastoris* [6] (Table 4). From a downstream processing point of view, working with a protein secreted into the culture medium would be preferred over working with inclusion bodies. We have succeeded in producing high levels of IFNα-2b and with further protease reduction and optimization of cultivation conditions production levels can likely be increased further. *T. reesei* has the potential to produce over 100 g/L of its own native enzymes. We will continue to develop the strains to reach expression levels for therapeutic proteins, which are close to the very high, natural secretion potential of *T. reesei*.

**Table 4:** Expression systems reported for IFNα-2b production

Organism	Production level	Comments	Reference
*E. coli*	Not reported	Intracellular	[2]
*E. coli*	3 g/L	Refolded from inclusion bodies and purified	[13]
*S. cerevisiae*	Not reported	Secreted	[3]
*B. subtilis*	Not reported	Secreted	[4]
*P. pastoris*	0.45 g/L	Secreted	[5]
*P. pastoris*	0.60 g/L	Secreted	[6]
*L. lactis*	2.4 µg/L	Intracellular/secreted	[7]
*Y. lipolytica*	0.43 g/L	Secreted	[8]
Mouse cells	0.12 g/L	Secreted	[9]
*T. reesei*	2.4 g/L	Secreted	This work
*T. reesei*	4.5 g/L	Secreted; protease inhibitors included	This work

## Additional file

10.1186/s12934-016-0508-5 Lists of primers used in PCR experiments.
10.1186/s12934-016-0508-5 Plasmid map of the *pep5* deletion vector, pTTv229.
10.1186/s12934-016-0508-5 The *kex2* expression cassette targeted to the *pep3* locus removed from plasmid pTTv205 by PmeI digestion.
10.1186/s12934-016-0508-5 Protease activity measurements of purified fractions from culture supernatant sampled on day 4 and 5. The fractions from the pepstatin columns were measured at pH 4.5 and the SBTI fractions at pH 5.5. The protease activity on day 5 was higher than on day 4.
10.1186/s12934-016-0508-5 Immunoblot detecting IFNα-2b produced by 9-protease deletion strains. Transformants from *amp1* (#1), *slp7* (#4, 13), *amp2* (#5-7), and *sep1* (#12) deletion strains were grown in duplicate in 24 well plates in TrMM with 8.6 g/L diammonium citrate, 5.4 g/L NaSO_4_, 100 mM PIPPS, 20 g/L spent grain extract, 40 g/L lactose at pH 5.5, shaking at 28 °C. The supernatant from day 6 was diluted so that 0.5 µl could be loaded per well. The M577 strain is the parental control (#8). The full length IFNα-2b runs around 17 kDa and the carrier bound material runs at 75 kDa.
10.1186/s12934-016-0508-5 Immunoblot detecting IFNα-2b production. A. 1 liter bioreactor cultures Triab116-118. The M669, M670, and M672 were grown in 40 g/L lactose, 20 g/L spent grain extract, 20 g/L whole spent grain, 5 g/L (NH_4_)_2_SO_4_, 5 g/L KH_2_PO_4_ at pH 4.5, 28 °C. The base level of the control M577 strain was 0.50 g/L. The supernatants were diluted so that 0.2 µl was loaded per well. IFNα-2b standards corresponding to 50, 100, 200 ng were loaded for quantification. B. 1 liter bioreactor cultures Triab119 and 121. The M673 and M674 strains were grown in same media. The supernatants were diluted so that 0.2 µl was loaded per well. The base level of the control M577 strain was 0.50 g/L. IFNα-2b standards corresponding to 50, 100, 200 ng were loaded for quantification.
10.1186/s12934-016-0508-5 Immunoblot detecting IFNα-2b production from bioreactor cultivations FTR108_R1 (M668 Δ*sep1*), FTR108_R4 (M671 Δ*pep9*), FTR108_R5 (M672 Δ*pep11*), FTR108_R6 (M673 Δ*slp7*), FTR108_R7 (M674 Δ*amp2*), and FTR109_R9 (M577). Strains were grown in 20 g/L yeast extract, 40 g/L cellulose, 80 g/L cellobiose, 40 g/L sorbose, pH 4.5. The supernatants were diluted so that 0.1 µl was loaded per well. Standard amounts of IFNα-2b corresponding to 50, 100, and 200 ng were used to generate a standard curve. The full length IFNα-2b runs around 17 kDa and the carrier bound material runs at 75 kDa.
10.1186/s12934-016-0508-5 Bioreactor data from cultivation of 9-protease deletion strains expressing IFNα-2b. Strains were grown in 20 g/L yeast extract, 40 g/L cellulose, 80 g/L cellobiose, 40 g/L sorbose, pH 4.5. Graphs show capacitance (top panel), base consumption (lower panel), and CO_2_ generation (both panels) as indicators for fungal growth. In the upper panel the capacitance data for M577 was not available and is not shown. In the figure legends dsep1 = Δ*sep1*, dpep9 = Δ*pep9*, etc. Growth of 9-protease deletion strains M671 (Δ*pep9*), M673 (Δ*slp7*), and M674 (Δ*amp2*) is comparable to M577 control, whereas M668 (Δ*sep1*) showed slightly elevated growth performance.

## Abbreviations

IFNα-2b: interferon alpha 2b
SBTI: soybean trypsin inhibitor
TrMM: *Trichoderma* minimal medium
