# Supplementary material for: Enabling low cost biopharmaceuticals: high level interferon alpha-2b production in Trichoderma reesei
Source: Microb Cell Fact. 2016 Jun 10;15:104. doi: 10.1186/s12934-016-0508-5 (PMC4902970; doi:10.1186/s12934-016-0508-5)
Supplement: Supplementary file 1 — 10.1186/s12934-016-0508-5 Lists of primers used in PCR experiments. [file 12934_2016_508_MOESM1_ESM.docx]

**Table S1: Primers for generating *pep5* deletion plasmid.**

| **Deletion plasmid pTTv202 for pep5 (TreID81004), vector backbone pRS426** | |
| --- | --- |
| Primer | Sequence |
| T372_pep5_5f_for | GGTAACGCCAGGGTTTTCCCAGTCACGACGGTTTAAACGGAGGCTGCGACACCGTCTG |
| T373_pep5_5f_rev | GCGCTGGCAACGAGAGCAGAGCAGCAGTAGTCGATGCTAGGCGGCCGCCCGGCCTGAAACGACCTCCC |
| T376_pep5_5DR_for | CCCGTCACCGAGATCTGATCCGTCACCGGGATCCACTTAAGCGGCCGCGAGAGAGAAACAAAACAGTG |
| T377_pep5_5DR_rev | ACATTCCGACCGTTTACTGATCCAAGCCGTGCAACCGACTGGCGCGCCCCGGCCTGAAACGACCTCCC |
| T378_pep5_3f_for | AGTCGGTTGCACGGCTTGGA |
| T379_pep5_3f_rev | AGCGGATAACAATTTCACACAGGAAACAGCGTTTAAACGAGACGGACGCCTGCACCAC |
| T374_bar_recpyr4_for2 | TGATTGTACCCCAGCTGCGATTGATGTGTATCTTTGCATGGCGATCGCGACAGAAGATGATATTGAAG |
| T375_bar_rev | TTAAGTGGATCCCGGTGACG |
|  |  |
| **Deletion plasmid pTTv229 for pep5 (TreID81004), vector backbone pTTv202** | |
| Primer | Sequence |
| no new primers, pTTv202 digested with NotI and ligated with pyr4 fragment from pTTv181 | |

**Table S2: Primers for generating *pep3* locus, *kex2* overexpression plasmid.**

| **Plasmid pTTv205 for *pep3* (TreID121133) locus, *kex2* (TreID123561), vector backbone pRS426** | |
| --- | --- |
| Primer | Sequence |
| vector_pep3_5f | GGTAACGCCAGGGTTTTCCCAGTCACGACGGTTTAAACGTCGAGCCCCCTGGACACCT |
| pep3_5f_rev | CATCGCCGTCGCGGACATGA |
| pep3_5f_cDNAfw | GCTGGCCGCTGGGAATAGCGTCATGTCCGCGACGGCGATGGAATTCGGTCTGAAGGACGT |
| pcDNA1_rev | GTTGAGAGAAGTTGTTGGAT |
| pcDNA1_kex2_fw | AACCAAAGACTTTTTGATCAATCCAACAACTTCTCTCAACATGAAGATTTCCTCGATCCT |
| kex2_rev_ | TCAGCGCCGTAACCTCTGCT |
| kex2_trpc_fw | TGATGGTGATGAGGCGGAAAAGCAGAGGTTACGGCGCTGAGGATCCACTTAACGTTACTG |
| trpc_rev | TCTCCTTCTAGAAAGAAGGA |
| pep3 3flankDR_F-trpCterm | GTACACTTGTTTAGAGGTAATCCTTCTTTCTAGAAGGAGAGCGGCCGCGGAGCCCTGGTCTGGTCCGT |
| pep3 3flankDR_rev | GCGCTGGCAACGAGAGCAGAGCAGCAGTAGTCGATGCTAGAAGCTGACGGGCGTCAACG |
| T094 pyr4_fw | TAGCATCGACTACTGCTGC |
| pyr4_rev-pep3 3flank | GTACGTTCTGATTGCCAACTACGGACCAGACCAGGGCTCCGCGGCCGCCATGCAAAGATACACATCAATC |
| T350_pep3_3f_fw | GGAGCCCTGGTCTGGTCCGT |
| T351_pep3_3f_rev | AGCGGATAACAATTTCACACAGGAAACAGCGTTTAAACACGCGCTTCAACATGCCCCA |
|  |  |
| **screening primers** |  |
| T354_pep3_5int | TCCCAAAGGAGCGCAACGGC |
| pcDNA1_rev | ACGTGGAATGATGGACTTAA |
| T355_pep3_3int | CGGAGCCCAGGCTCTGACCA |
| T028_pyr4_rev | CATCCTCAAGGCCTCAGAC |

**Table S3: Primers for screening removal of *pyr4* cassette from 7-fold strain and for screening *pep5* integration and strain purity.**

| **For screening removal of *pyr4* cassette in *pep3* locus** | |
| --- | --- |
| Primer | Sequence |
| T047_trpC_term_end_F | CCTATGAGTCGTTTACCCAGA |
| T854_pep3_3f_r2 | TGGCCGAGTCTATGCGTA |
| T488_pyr4_5utr_rev | GGAGTTGCTTTAATGTCGGG |
| T061_pyr4_orf_screen_2F | TTAGGCGACCTCTTTTTCCA |
| T855_pep3_orf_f3 | GTAAGACGCCCCGTCTC |
| T754_pep3_orf_rev2 | TGGATCATGTTGGCGACG |
|  |  |
| **For screening integration of pTTv229** | |
| Primer | Sequence |
| T627_pep5_5int_new | GTCGAAGATGTCCTCGAGAT |
| T488_pyr4_5utr_rev | GGAGTTGCTTTAATGTCGGG |
| T061_pyr4_orf_screen_2F | TTAGGCGACCTCTTTTTCCA |
| T628_pep5_3int_new | TAGTCCATGCCGAACTGC |
|  |  |
| **For screening deletion of *pep5* ORF** | |
| Primer | Sequence |
| T418_pep5_orf_for | CCGGACCTGCACCGCAAGTT |
| T419_pep5_orf_rev | AGGGCAATGTCGCCCAGCAC |
| T859_pep5_orf_f2 | GACCTGCACCGCAAGTT |
| T860_pep5_orf_f3 | GTCGAGCGTCTGATATTCAC |
| T861_pep5_orf_r2 | GACGGAGACCTCCCACA |

###

### Table S4. Primers for screening integration into the *cbh1* locus

| **5’ integration, T095 + T096, ~2.8 kb (2776 bp), 58°C** |
| --- |
| T095_F_cbhI                                                              GCTGTTCCTACAGCTCTTTC |
| T096_R_cbhI_exon                                              AGCCGCACGGCAGC |
|  |
|  |
| **3’ integration, T008 + T022, ~1.9 kb, 60°C** |
| T008_pHHO1-CBHIloc_cbh13'flankOutRev                               GGTTGACTTACTCCAGATCG |
| T022_Amds_start_rc_seg                                                             CTGAAGCAACAGGTGCCAAG |
|  |
|  |
| **ORF, T1720+T1721, gives a PCR band of 770 bp if the *cbh1* gene is not deleted. Pure transformants do not give signal. 68°C** |
| T1720_cbh1intronfor                                                                       CCTGACGCTATCTTCTTGTTGG |
| T1721_cbh1intronrev                                                                       CGCGCATGTTTGTCCATCAAAC |

### Southern blotting for IFN-α 2b expressing strains

In order to check that our strain expressing IFN-α 2b carries only one copy of expression vector we carried out Southern blot analyses using traditional ^32^P-dCTP radioactive labelling method. Strains were cultivated for DNA extraction in shake flasks. Genomic DNA was extracted using Easy-DNA kit (Invitrogen). gDNA was digested with *XhoI* for IFN-α 2b probe and with *PstI* for 5’ and 3’ flank probes. Positive control plasmid was digested with *Mss*I for IFN-α 2b probe and for 5’ and 3’ flank probes.

**Table S5. Primers used to produce probes for Southern analyses of *IFN-α 2b expressing strains*.**

| **Probe** | **Primer** | **Sequence** | **Probe size** |
| --- | --- | --- | --- |
| IFN-alpha 2b | T888_IFN_GGGGG-NVISKR_fwd  T889_IFN-alpha_tcbh1_rev | CACCACCACCCGCCGCCCAGCCACTACCACTGGAAGCTCTGGCGGCGGCGGCGGCAACGTCATCAGCAAGCGCTGCGACCTCCCCCAGACCCA  CCAAGAATCTACCGGTGCGTCAGGCTTTCGCCACGGAGCTTCATTCCTTGCTGCGGAGGC | 611 bp |
| 5’ flank | T1679  T173_pcbh1_seq_r1 | CAACCTTTGGCGTTTCCCTG  CAAAGGCCGAAGGCCCGAGG | 799 bp |
| 3’ flank | T178_cbh13flank_seq_f2  T1680 | GGCCGCAGGCCCATAACCAG  TGAGTGGGGGATGACAGACA | 796 bp |

**Table S6: Primers for generating *pep8* deletion plasmid.**

| **Deletion plasmid pTTv266 (Δ*pep8-pyr4-hgh*), vector backbone pRS426** | |
| --- | --- |
| Primer | Sequence |
| T1019_pep8_5flkfw_vector | GTAACGCCAGGGTTTTCCCAGTCACGACGGTTTAAACAGGTTTGGGTTGTGAGATCG |
| T1020_pep8_5flkrev_pyr4Prom | GCGCTGGCAACGAGAGCAGAGCAGCAGTAGTCGATGCTAGGCGGCCGCGCGCAAAGCTACTGGGCTAT |
| T1021_pep8_3flkfw_pyr4loop | CAACCAGCCGCAGCCTCAGCCTCTCTCAGCCTCATCAGCCGCGGCCGCTGGCTTTTCTCAGCCCTATG |
| T1022_pep8_3flkrev_vector | GCGGATAACAATTTCACACAGGAAACAGCGTTTAAACCAATGTGTGCCTGTTTTTCG |

**Table S7: Primers for screening pTTv266/Δ*pep8-pyr4-hgh* integration and strain purity.**

|  |  |
| --- | --- |
| **For screening integration of pTTv266 (Δ*pep8-pyr4-hgh*)** | |
| Primer | Sequence |
| T1023_pep8_screen_5flk_fwd | TTTATCCGCTTCCACGACAC |
| T1084_screen_5flk_pyr_rev | TCTTGAGCACGACAATCGAC |
| T1015_screen_3flk_hygro_fwd | GCATGGTTGCCTAGTGAATG |
| T1024_pep8_screen_3flk_rev | CGATGGTGAAGTCAATGTGG |
|  |  |
| **For screening deletion of *pep8* ORF** | |
| T1025_pep8_orf_fwd | GGCGATTACTTCCAGGACAA |
| T1026_pep8_orf_rev | CAATGGCAATCTGGTTGTTG |

**Table S8: Primers for generating *pep9* deletion plasmid.**

| **Deletion plasmid pTTv267 (Δ*pep9-pyr4-hgh*), vector backbone pRS426** | |
| --- | --- |
| Primer | Sequence |
| T1027_pep9_5flkfw_vector | GTAACGCCAGGGTTTTCCCAGTCACGACGGTTTAAACAACCACGACGAAAATCAAGG |
| T1028_pep9_5flkrev_pyr4Prom | GCGCTGGCAACGAGAGCAGAGCAGCAGTAGTCGATGCTAGGCGGCCGCAATGGACCCAGATGTCAAGG |
| T1029_pep9_3flkfw_pyr4loop | CAACCAGCCGCAGCCTCAGCCTCTCTCAGCCTCATCAGCCGCGGCCGCGATCTAGGATTCGCCAAACG |
| T1030_pep9_3flkrev_vector | GCGGATAACAATTTCACACAGGAAACAGCGTTTAAACACGACATGAACAAACGGACA |

**Table S9: Primers for screening pTTv267/Δ*pep9-pyr4-hgh* integration and strain purity.**

|  |  |
| --- | --- |
| **For screening integration of pTTv267 (Δ*pep9-pyr4-hgh*)** | |
| Primer | Sequence |
| T1031_pep9_screen_5flk_fwd | GGGTTGGAGATGTTGGAAGA |
| T1084_screen_5flk_pyr_rev | TCTTGAGCACGACAATCGAC |
| T1015_screen_3flk_hygro_fwd | GCATGGTTGCCTAGTGAATG |
| T1032_pep9_screen_3flk_rev | TTGACGAGACGGGGAGTTAC |
|  |  |
| **For screening deletion of *pep9* ORF** | |
| T1033_pep9_orf_fwd | CAGCCCTGACACCACTCTCT |
| T1034_pep9_orf_rev | TCCAGTCCTTGGGAGAAATG |

**Table S10: Primers for generating *pep11* deletion plasmids.**

| **Deletion plasmid pTTv268 (Δ*pep11-pyr4-hgh*), vector backbone pRS426** | |
| --- | --- |
| Primer | Sequence |
| T1009_pep11_5flkfw_vector | GTAACGCCAGGGTTTTCCCAGTCACGACGGTTTAAAC  ATGAGCGTGATCGACAAGTG |
| T1010_pep11_5flkrev_pyr4Prom | GCGCTGGCAACGAGAGCAGAGCAGCAGTAGTCGATGCTAG  GCGGCCGCCCTCTGAGGTCGAGATGGAG |
| T1011_pep11_3flkfw_pyr4loop | CAACCAGCCGCAGCCTCAGCCTCTCTCAGCCTCATCAGCCGCGGCCGCTTTGCATGTGAATACGAGATGA |
| T1012_pep11_3flkrev_vector | GCGGATAACAATTTCACACAGGAAACAGCGTTTAAAC  TGCTCGATCCTACTCCAAGG |

**Table S11: Primers for screening pTTv268/Δ*pep11-pyr4-hgh* integration and strain purity.**

|  |  |
| --- | --- |
| **For screening integration of pTTv268 (Δ*pep11-pyr4-hgh*)** | |
| Primer | Sequence |
| T1013_pep11_screen_5flk_fwd | TTACGACTCGATCCCTGTCC |
| T1084_screen_5flk_pyr_rev | TCTTGAGCACGACAATCGAC |
| T1015_screen_3flk_hygro_fwd | GCATGGTTGCCTAGTGAATG |
| T1016_pep11_screen_3flk_rev | GCCGCTAGGATCGTGATAAG |
|  |  |
| **For screening deletion of *pep11* ORF** | |
| T1017_pep11_orf_fwd | GTGTCCCAGGACGACAACTT |
| T1018_pep11_orf_rev | TGAAGGTTGCAGTGATCTCG |

**Table S12: Primers for generating *amp1* deletion plasmid.**

| **Deletion plasmid pTTv240 (Δ*amp1-pyr4-hgh*), vector backbone pRS426** | |
| --- | --- |
| Primer | Sequence |
| T832_amp1_5flkfw_vector | GTAACGCCAGGGTTTTCCCAGTCACGACGGTTTAAACCATGGAAGATGCGAGCTACA |
| T833_amp1_5flkrev_pyr4Prom | GCGCTGGCAACGAGAGCAGAGCAGCAGTAGTCGATGCTAGGCGGCCGCGGAGAGGAGATGGGTGTTGA |
| T836_amp1_3flkfw_5DR_end | CCCCCCTTTCTCTCTCTCTTTCAACACCCATCTCCTCTCCGGCGCGCCGCGAGGTGCGTTTCTGTAGC |
| T837_amp1_3flkrev_vector | GCGGATAACAATTTCACACAGGAAACAGCGTTTAAACCGGCAAATACTACGACGACA |
| T834_amp1_5 DR fwd | GTACACTTGTTTAGAGGTAATCCTTCTTTCTAGAAGGAGAGCGGCCGCGTCGAGTGCATCAATGACGA |
| T835_amp1_5 DR rev | CAAACAGCATGCTCGTAAATGCTACAGAAACGCACCTCGCGGCGCGCCGGAGAGGAGATGGGTGTTGA |

**Table S13: Primers for screening pTTv240/Δ*amp1-pyr4-hgh* integration and strain purity.**

|  |  |
| --- | --- |
| **For screening integration of pTTv240 (Δ*amp1-pyr4-hgh*)** | |
| Primer | Sequence |
| T840_amp1_screen_5 flk fwd | TGGCATTGATCTAGAACCTCCT |
| T1084_screen_5flk_pyr_rev | TCTTGAGCACGACAATCGAC |
| T1015_screen_3flk_hygro_fwd | GCATGGTTGCCTAGTGAATG |
| T843_amp1_scrn_rev 3 flk | GACGACTTGGTGGAGCTCAT |
|  |  |
| **For screening deletion of *amp1* ORF** | |
| T940_amp1_orf2_fw | GACTACCCCCAGAACGTCAA |
| T941_amp1_orf2_rev | AAGAGGCGGATCTTTTGGTT |

**Table S14: Primers for generating *slp7* deletion plasmids.**

| **Deletion plasmid pTTv269 (Δ*pep11-pyr4-hgh*), vector backbone pRS426** | |
| --- | --- |
| Primer | Sequence |
| T1088_slp7_5flkfw_vector | GTAACGCCAGGGTTTTCCCAGTCACGACGGTTTAAACTCCCATATGCCTCTTGAAGG |
| T1089_slp7_5flkrev_pyr4Prom | GCGCTGGCAACGAGAGCAGAGCAGCAGTAGTCGATGCTAGGCGGCCGCTTTGCAGCAAGATGTCGTTC |
| T1090_slp7_3flkfw_pyr4loop | CAACCAGCCGCAGCCTCAGCCTCTCTCAGCCTCATCAGCCGCGGCCGCTGGGTGATAAGCTTGGGTTT |
| T1091_slp7_3flkrev_vector | GCGGATAACAATTTCACACAGGAAACAGCGTTTAAACATCATGATGACCCATCGACA |

**Table S15: Primers for screening pTTv269/Δ*slp7-pyr4-hgh* integration and strain purity.**

|  |  |
| --- | --- |
| **For screening integration of pTTv269 (Δ*slp7-pyr4-hgh*)** | |
| Primer | Sequence |
| T1092_slp7_screen_5flk_fwd | TTGGTTTGAACAGCTGCAAG |
| T1084_screen_5flk_pyr_rev | TCTTGAGCACGACAATCGAC |
| T1015_screen_3flk_hygro_fwd | GCATGGTTGCCTAGTGAATG |
| T1093_slp7_screen_3flk_rev | ATGGTCAGCCAGAACCTGAC |
|  |  |
| **For screening deletion of *slp7* ORF** | |
| T1094_slp7_orf_fwd | TCTTGAGCCGTTTCTCGAAT |
| T1095_slp7_orf_rev | CCGCTCTTAGATCGATGGTC |

**Table S16: Primers for generating *amp2* deletion plasmids.**

| **Deletion plasmid pTTv271 (Δ*amp2-pyr4-hgh*), vector backbone pRS426** | |
| --- | --- |
| Primer | Sequence |
| T1079_amp2_5flkfw_vector | GTAACGCCAGGGTTTTCCCAGTCACGACGGTTTAAACCCATTCTCGTCGTTGTTTCC |
| T1080_amp2_5flkrev_pyr4Prom | GCGCTGGCAACGAGAGCAGAGCAGCAGTAGTCGATGCTAGGCGGCCGCTGGAGGAGTAGCTGCACTGA |
| T1081_amp2_3flkfw_pyr4loop | CAACCAGCCGCAGCCTCAGCCTCTCTCAGCCTCATCAGCCGCGGCCGCACAGCCAGTGGAAACCAAAC |
| T1082_amp2_3flkrev_vector | GCGGATAACAATTTCACACAGGAAACAGCGTTTAAACTAGAGCTTGGAGGGAACAGG |

**Table S17: Primers for screening pTTv271/Δ*amp2-pyr4-hgh* integration and strain purity.**

|  |  |
| --- | --- |
| **For screening integration of pTTv271 (Δ*amp2-pyr4-hgh*)** | |
| Primer | Sequence |
| T1083_amp2_screen_5flk_fwd | CCACTGAAGGGGAGTTTTCA |
| T1084_screen_5flk_pyr_rev | TCTTGAGCACGACAATCGAC |
| T1015_screen_3flk_hygro_fwd | GCATGGTTGCCTAGTGAATG |
| T1085_amp2_screen_3flk_rev | TCGCGGTATCGTATGAGATG |
|  |  |
| **For screening deletion of *amp2* ORF** | |
| T1086_amp2_orf_fwd | GCCAGCTTCAACATCGACTT |
| T1087_amp2_orf_rev | CAGCACGAGCACGTTGTACT |

**Table S18: Primers for generating *sep1* deletion plasmid.**

| **Deletion plasmid pTTv247 (Δ*sep1-pyr4-hgh*), vector backbone pRS426** | |
| --- | --- |
| Primer | Sequence |
| T489_serendo_5f_for | GGTAACGCCAGGGTTTTCCCAGTCACGACGGTTTAAACATGGGCTGAAACCGGCGCAA |
| T490_serendo_5f_rev | GCGCTGGCAACGAGAGCAGAGCAGCAGTAGTCGATGCTAGGCGGCCGCGACAGCGCCTCGCCAAGTGT |
| T498_serendo_3f_for | ATGATGCCTTTGCAGAAATGGCTTGCTCGCTGACTGATACGGCGCGCCTGGCGCTTCCGTTCCCTTCC |
| T499_serendo_3f_rev | AGCGGATAACAATTTCACACAGGAAACAGCGTTTAAACTGTTGAGACGGGCGAGTGCT |
| T1000_serendo_5dr_for2 | TGATTGTACCCCAGCTGCGATTGATGTGTATCTTTGCATGGCGGCCGCAGCAGCCTGCCCAGAGAATC |
| T1001_serendo_5dr_rev2 | GACAATCAGAGGCCTCAATTGGAAGGGAACGGAAGCGCCAGGCGCGCCGACAGCGCCTCGCCAAGTGT |

**Table S19: Primers for screening pTTv247/Δ*sep1-pyr4-hgh* integration and strain purity.**

|  |  |
| --- | --- |
| **For screening integration of pTTv247 (Δ*sep1-pyr4-hgh*)** | |
| Primer | Sequence |
| T519_serendo_5int | AACCACCTTGTTCTGTCCGT |
| T488_pyr4_5utr_rev | GGAGTTGCTTTAATGTCGGG |
| T521_serendo_3int | GGAACTGTCAAGATCTGGGA |
| T1015_screen_3flk_hygro_fwd | GCATGGTTGCCTAGTGAATG |
|  |  |
| **For screening deletion of *sep1* ORF** | |
| T504_serendo_orf_probef | GCCTCCGCCCTCCTCTTCCA |
| T505_serendo_orf_prober | GCTTTGTCGAGCGGAGCGGT |

**Table S20: Primers for generating *mep1* deletion plasmids.**

| **Deletion plasmid pTTv270 (Δ*mep1-pyr4-hgh*), vector backbone pRS426** | |
| --- | --- |
| Primer | Sequence |
| T1096_mp1_5flkfw_vector | GTAACGCCAGGGTTTTCCCAGTCACGACGGTTTAAACTGAAGTCGTCTGCAAAGTCG |
| T1097_mp1_5flkrev_pyr4Prom | GCGCTGGCAACGAGAGCAGAGCAGCAGTAGTCGATGCTAGGCGGCCGCGATGGTCAATTGAGCGGTTT |
| T1098_mp1_3flkfw_pyr4loop | CAACCAGCCGCAGCCTCAGCCTCTCTCAGCCTCATCAGCCGCGGCCGCGATGCGAAGCGAATGGAG |
| T1099_mp1_3flkrev_vector | GCGGATAACAATTTCACACAGGAAACAGCGTTTAAACGCAAATGCCGTCAAGGTC |

**Table S21: Primers for screening pTTv270/Δ*mep1-pyr4-hgh* integration and strain purity.**

|  |  |
| --- | --- |
| **For screening integration of pTTv270 (Δ*mep1-pyr4-hgh*)** | |
| Primer | Sequence |
| T1100_mp1_screen_5flk_fwd | GTCTTGGCCATCAATGGAGT |
| T1084_screen_5flk_pyr_rev | TCTTGAGCACGACAATCGAC |
| T1015_screen_3flk_hygro_fwd | GCATGGTTGCCTAGTGAATG |
| T1101_mp1_screen_3flk_rev | ACGGCTTACGAACAACGAGT |
|  |  |
| **For screening deletion of *mep1* ORF** | |
| T1102_mp1_orf_fwd | ACATCCTGGCCGATATTCTG |
| T1103_mp1_orf_rev | GCTGTAGCTGGTGGAGAAGC |
