# Supplementary figures and images for: Enabling low cost biopharmaceuticals: high level interferon alpha-2b production in Trichoderma reesei
Source: Microb Cell Fact. 2016 Jun 10;15:104. doi: 10.1186/s12934-016-0508-5 (PMC4902970; doi:10.1186/s12934-016-0508-5)

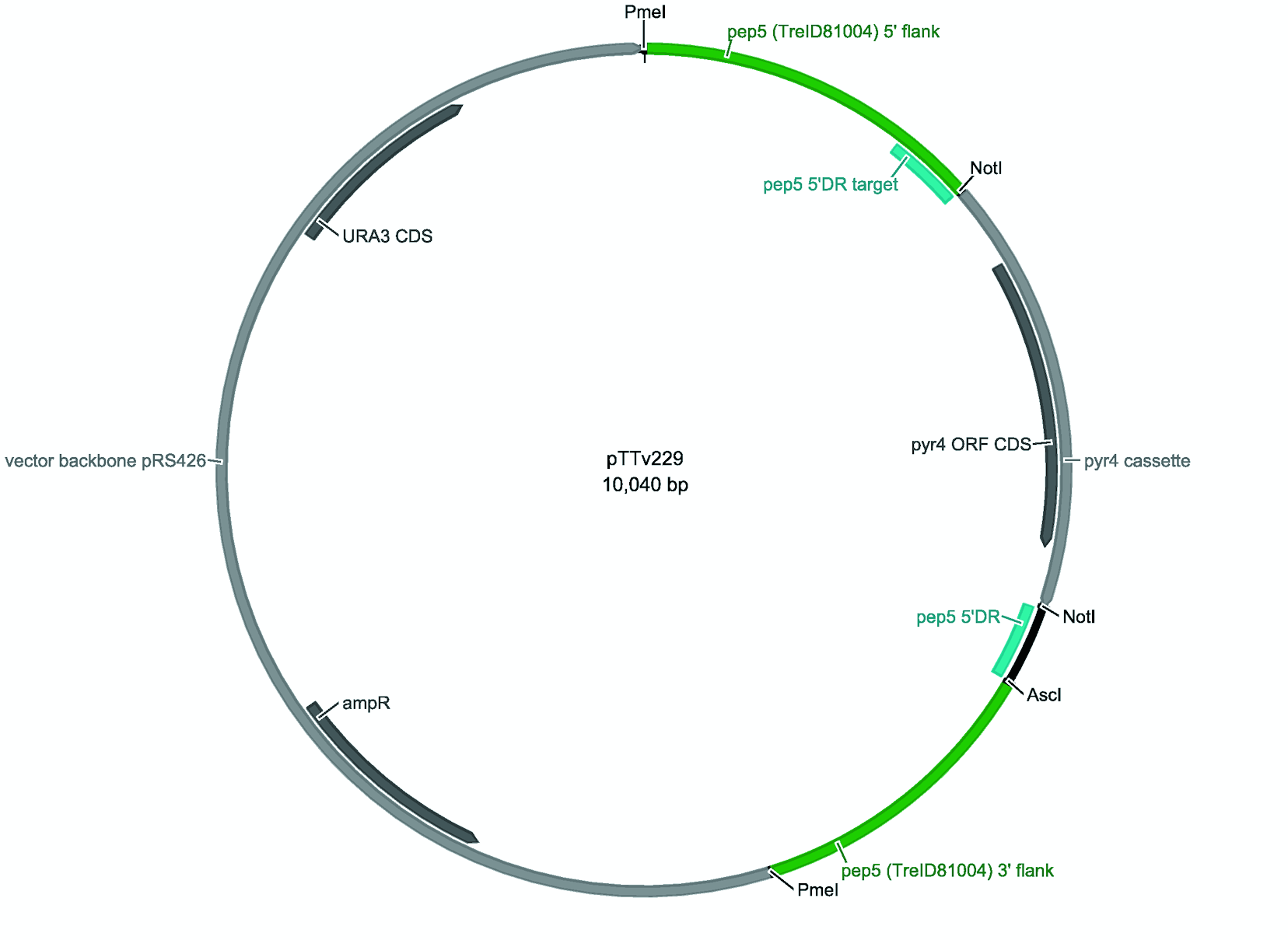

Supplement: Supplementary file 2 — 10.1186/s12934-016-0508-5 Plasmid map of the pep5 deletion vector, pTTv229. [file 12934_2016_508_MOESM2_ESM.tif]

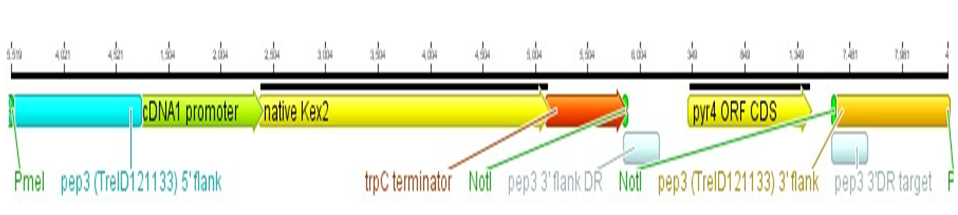

Supplement: Supplementary file 3 — 10.1186/s12934-016-0508-5 The kex2 expression cassette targeted to the pep3 locus removed from plasmid pTTv205 by PmeI digestion. [file 12934_2016_508_MOESM3_ESM.tif]

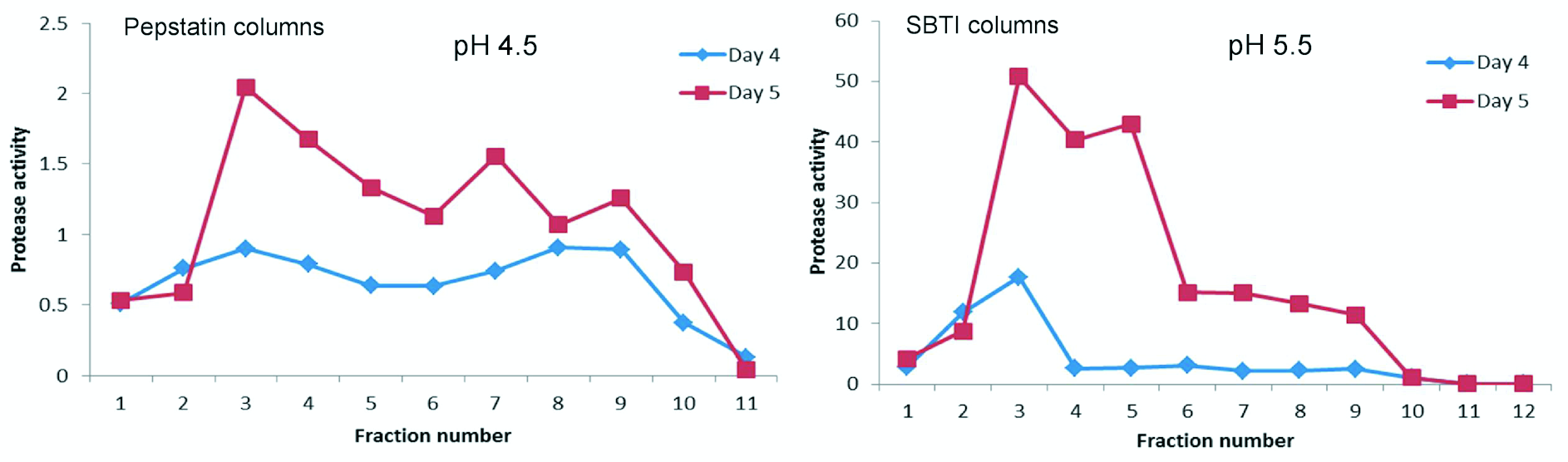

Supplement: Supplementary file 4 — 10.1186/s12934-016-0508-5 Protease activity measurements of purified fractions from culture supernatant sampled on day 4 and 5. The fractions from the pepstatin columns were measured at pH 4.5 and the SBTI fractions at pH 5.5. The protease activity on day 5 was higher than on day 4. [file 12934_2016_508_MOESM4_ESM.tif]

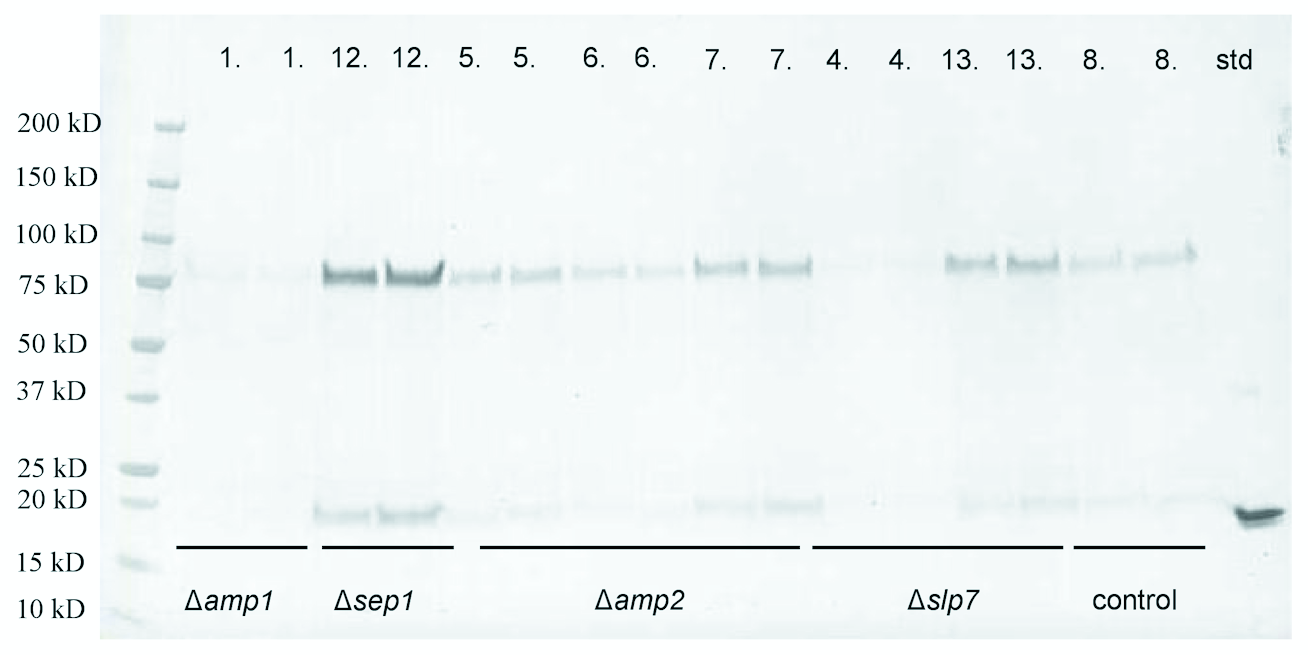

Supplement: Supplementary file 5 — 10.1186/s12934-016-0508-5 Immunoblot detecting IFNα-2b produced by 9-protease deletion strains. Transformants from amp1 (#1), slp7 (#4, 13), amp2 (#5-7), and sep1 (#12) deletion strains were grown in duplicate in 24 well plates in TrMM with 8.6 g/L diammonium citrate, 5.4 g/L NaSO4, 100 mM PIPPS, 20 g/L spent grain extract, 40 g/L lactose at pH 5.5, shaking at 28 °C. The supernatant from day 6 was diluted so that 0.5 µl could be loaded per well. The M577 strain is the parental control (#8). The full length IFNα-2b runs around 17 kDa and the carrier bound material runs at 75 kDa. [file 12934_2016_508_MOESM5_ESM.tif]

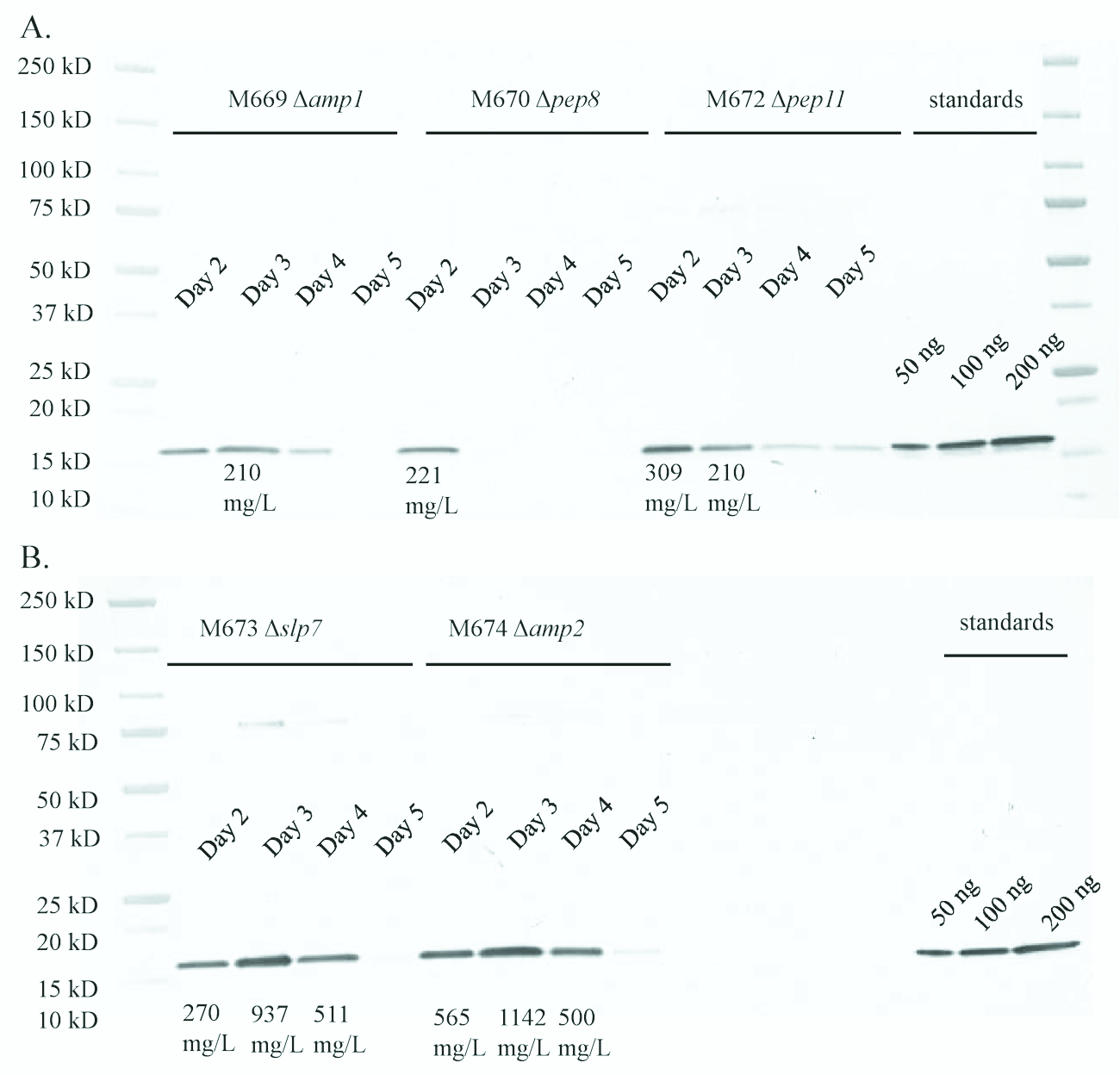

Supplement: Supplementary file 6 — 10.1186/s12934-016-0508-5 Immunoblot detecting IFNα-2b production. A. 1 liter bioreactor cultures Triab116-118. The M669, M670, and M672 were grown in 40 g/L lactose, 20 g/L spent grain extract, 20 g/L whole spent grain, 5 g/L (NH4)2SO4, 5 g/L KH2PO4 at pH 4.5, 28 °C. The base level of the control M577 strain was 0.50 g/L. The supernatants were diluted so that 0.2 µl was loaded per well. IFNα-2b standards corresponding to 50, 100, 200 ng were loaded for quantification. B. 1 liter bioreactor cultures Triab119 and 121. The M673 and M674 strains were grown in same media. The supernatants were diluted so that 0.2 µl was loaded per well. The base level of the control M577 strain was 0.50 g/L. IFNα-2b standards corresponding to 50, 100, 200 ng were loaded for quantification. [file 12934_2016_508_MOESM6_ESM.tif]

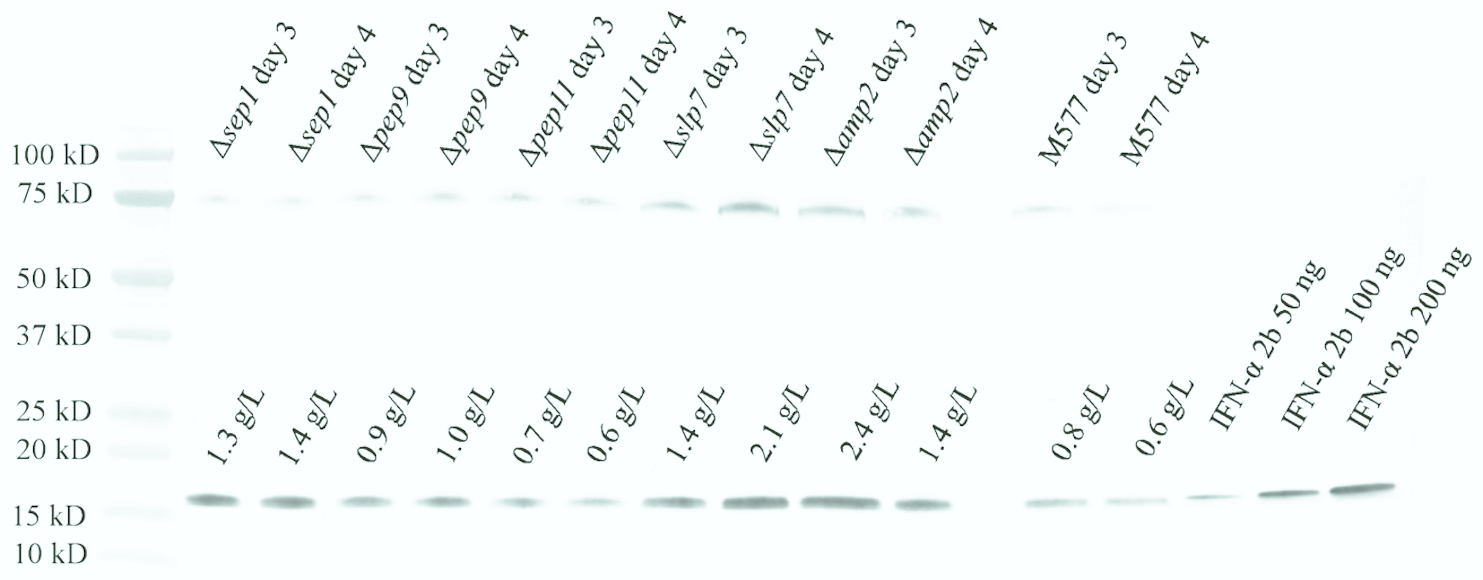

Supplement: Supplementary file 7 — 10.1186/s12934-016-0508-5 Immunoblot detecting IFNα-2b production from bioreactor cultivations FTR108_R1 (M668 Δsep1), FTR108_R4 (M671 Δpep9), FTR108_R5 (M672 Δpep11), FTR108_R6 (M673 Δslp7), FTR108_R7 (M674 Δamp2), and FTR109_R9 (M577). Strains were grown in 20 g/L yeast extract, 40 g/L cellulose, 80 g/L cellobiose, 40 g/L sorbose, pH 4.5. The supernatants were diluted so that 0.1 µl was loaded per well. Standard amounts of IFNα-2b corresponding to 50, 100, and 200 ng were used to generate a standard curve. The full length IFNα-2b runs around 17 kDa and the carrier bound material runs at 75 kDa. [file 12934_2016_508_MOESM7_ESM.tif]

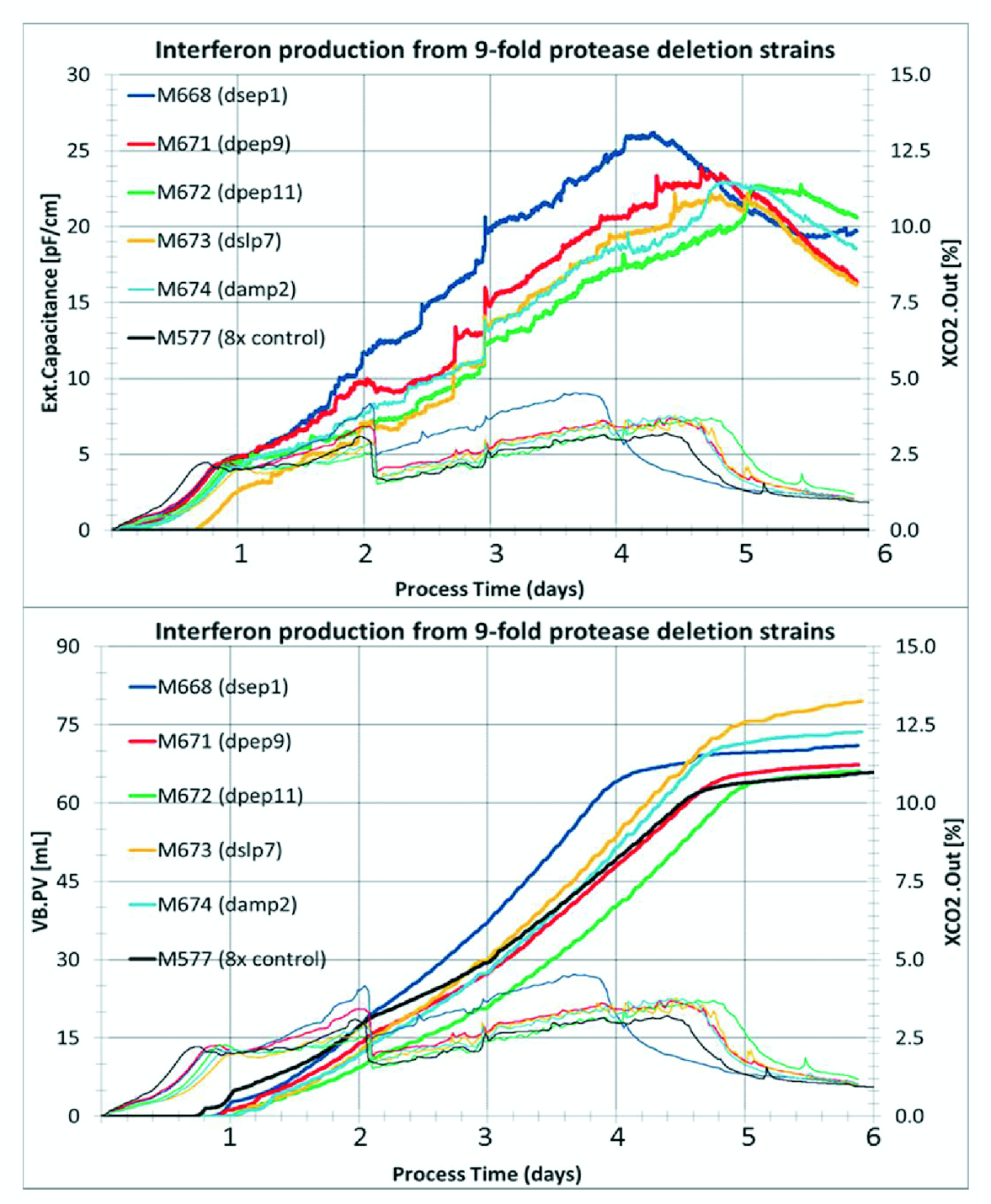

Supplement: Supplementary file 8 — 10.1186/s12934-016-0508-5 Bioreactor data from cultivation of 9-protease deletion strains expressing IFNα-2b. Strains were grown in 20 g/L yeast extract, 40 g/L cellulose, 80 g/L cellobiose, 40 g/L sorbose, pH 4.5. Graphs show capacitance (top panel), base consumption (lower panel), and CO2 generation (both panels) as indicators for fungal growth. In the upper panel the capacitance data for M577 was not available and is not shown. In the figure legends dsep1 = Δsep1, dpep9 = Δpep9, etc. Growth of 9-protease deletion strains M671 (Δpep9), M673 (Δslp7), and M674 (Δamp2) is comparable to M577 control, whereas M668 (Δsep1) showed slightly elevated growth performance. [file 12934_2016_508_MOESM8_ESM.tif]
